# New results on the continuous Weinstein wavelet transform

**DOI:** 10.1186/s13660-017-1534-5

**Published:** 2017-10-28

**Authors:** Hatem Mejjaoli, Ahmedou Ould Ahmed Salem

**Affiliations:** 1 College of Sciences, Department of Mathematics, Taibah University, PO BOX 30002, Al Madinah Al Munawarah, Saudi Arabia; 2 College of Sciences, Department of Mathematics, King Khalid University, Mohayil, Saudi Arabia

**Keywords:** 44A05, 42B10, Weinstein operator, Weinstein wavelet transform, localization operators, Schatten-von Neumann class, Heisenberg’s type inequalities

## Abstract

We consider the continuous wavelet transform $\mathcal{S}_{h}^{W}$ associated with the Weinstein operator. We introduce the notion of localization operators for $\mathcal {S}_{h}^{W}$. In particular, we prove the boundedness and compactness of localization operators associated with the continuous wavelet transform. Next, we analyze the concentration of $\mathcal{S}_{h}^{W}$ on sets of finite measure. In particular, Benedicks-type and Donoho-Stark’s uncertainty principles are given. Finally, we prove many versions of Heisenberg-type uncertainty principles for $\mathcal{S}_{h}^{W}$.

## Introduction

In this paper, we consider the Weinstein operator (also called the Laplace-Bessel differential operator (see [1])) defined on $\Bbb{R}^{d-1}\times (0,\infty)$ by

$$\begin{aligned} \triangle_{\beta} &:=\sum_{i=1}^{d} \frac{\partial^{2}}{\partial x_{i}^{2}}+\frac{2\beta+1}{x_{d}}\frac{\partial}{\partial x_{d}} \\ &=\triangle_{x'}+\mathcal{L} _{\beta,x_{d}},\quad\beta>- \frac{1}{2}, \end{aligned}$$

where $\triangle_{x'}$ is the Laplace operator on $\mathbb {R}^{d-1}$, and $\mathcal{L}_{\beta,x_{d}}$ the Bessel operator on $(0,\infty)$ given by

1.1 $$ \mathcal{L}_{\beta,x_{d}} := \frac{d^{2}}{dx_{d}^{2}} + \frac{2\beta+ 1}{x_{d}} \frac{d}{dx_{d}}, \quad\beta> -\frac{1}{2}. $$

The Weinstein operator $\triangle_{\beta}$ has several applications in pure and applied mathematics especially in fluid mechanics (see [2]).

The harmonic analysis associated with the Weinstein operator is studied by Ben Nahia and Ben Salem [3, 4]. In particular the authors have introduced and studied the generalized Fourier transform associated with the Weinstein operator. This transform is called the Weinstein transform.

In the classical setting, the notion of wavelets was first introduced by Morlet, a French petroleum engineer at ELF-Aquitaine, in connection with his study of seismic traces. The mathematical foundations were given by Grossmann and Morlet [5]. The harmonic analyst Meyer and many other mathematicians became aware of this theory, and they recognized many classical results inside it (see [6–8]). Classical wavelets have wide applications, ranging from signal analysis in geophysics and acoustics to quantum theory and pure mathematics (see [9–11] and the references therein).

Next, the theory of wavelets and continuous wavelet transforms has been extended to hypergroups, in particular, to the Chébli-Trimèche hypergroups (see [12]).

Recently in [13] the authors have introduced and studied the Weinstein wavelet transform $\mathcal{S}_{h}^{W}$. In the same paper, for the transform $\mathcal{S}_{h}^{W}$, the authors have proved the Plancherel and inversion formulas.

Nowadays, the continuous wavelet transform is one of the useful subjects in harmonic analysis. In this paper we present only two subjects.

The first subject is the new uncertainty principles involving time-frequency representations.

The second subject is the localization operators. These operators were initiated by Daubechies [14–16], and detailed in the book [17] by Wong.

As the harmonic analysis associated with the Weinstein operator has known remarkable development, it is a natural question whether there exist an equivalent of the theory of localization operators and new uncertainty principles for the continuous wavelet transform relating to this harmonic analysis.

The purpose of the present paper is twofold. On one hand, we want to study many versions of quantitative uncertainty principles for the continuous Weinstein wavelet transform. On the other hand, we want to study the localization operators for the continuous Weinstein wavelet transform.

The remainder of this paper is arranged as follows. In Section 2, we recall the main results about the harmonic analysis associated with the Weinstein operator. In Section 3, we introduce and study the two-localization operators associated with the Weinstein continuous wavelet transform. More precisely, the Schatten-von Neumann properties of these two-wavelet localization operators are established, and for trace class localization operators, the traces and the trace class norm inequalities are presented. In Section 4, we study the quantitative analysis of the continuous Weinstein wavelet transform and time-frequency concentration. In particular, we give results on sets of finite measure and Donoho-Stark and Benedicks-type uncertainty principles. In Sections 5 and 6, we prove many versions of the Heisenberg uncertainty inequalities for the Weinstein continuous wavelet transform. Finally, some conclusions are drawn in Section 7.

## Preliminaries

To confirm the basic and standard notations, we briefly overview the Weinstein operator and related harmonic analysis. The main references are [3, 4].

### Harmonic analysis associated with the Weinstein operator

In this subsection, we collect some notation and results on the Weinstein kernel, the Weinstein transform, and the Weinstein convolution.

We use the following notation:

- $\overline{\mathbb {R}^{d}_{+}} = \mathbb {R}^{d-1} \times[0,\infty)$;
- $x = (x_{1},\ldots,x_{d-1},x_{d})=(x',x_{d}) \in\overline{\mathbb {R}^{d}_{+}}$;
- $C_{*}(\mathbb {R}^{d})$ is the space of continuous functions on $\mathbb {R}^{d}$ even with respect to the last variable.
- $C_{*}^{p}(\mathbb {R}^{d})$ is the space of functions of class $C^{p}$ on $\mathbb {R}^{d}$ even with respect to the last variable.
- $\mathcal{E}_{*}(\mathbb {R}^{d})$ is the space of $C^{\infty}$-functions on $\mathbb {R}^{d}$ even with respect to the last variable.
- $\mathcal{S}_{*}(\mathbb {R}^{d})$ is the Schwartz space of rapidly decreasing functions on $\mathbb {R}^{d}$ even with respect to the last variable.
- $D_{*}(\mathbb {R}^{d})$ is the space of $C^{\infty}$-functions on $\mathbb {R}^{d}$ with compact support and even with respect to the last variable.

The Weinstein kernel Λ is given by

2.1 $$ \Lambda(x,z) := e^{i\langle x',z'\rangle} j_{\beta}(x_{d}z_{d}) \quad{\text{for all }} (x,z) \in \mathbb {R}^{d}\times \mathbb {C}^{d}, $$

where $j_{\beta}$ is the normalized Bessel function. The Weinstein kernel satisfies the following properties:

(i) For all $z, t \in \mathbb {C}^{d}$, we have
  2.2 $$ \begin{gathered} \Lambda(z,t) = \Lambda(t,z), \qquad\Lambda(z,0) = 1, \quad{ \text{and}} \\ \Lambda(\lambda z,t) = \Lambda(z, \lambda t) \quad{\text{for all }} \lambda\in \mathbb {C}. \end{gathered} $$
(ii) For all $\nu\in \mathbb {N}^{d}$, $x \in \mathbb {R}^{d}$, and $z \in \mathbb {C}^{d}$, we have
  2.3 $$ \big|D_{z}^{\nu} \Lambda(x , z) \big| \leq\|x\|^{|\nu|} \exp \bigl( \|x\| \| \operatorname{Im}z\| \bigr), $$
  where $D_{z}^{\nu} = \frac{\partial^{\nu}}{\partial z_{1}^{\nu_{1}}\cdots\partial z_{d}^{\nu_{d}}}$ and $|\nu| = \nu_{1} + \cdots+ \nu_{d}$. In particular,
  2.4 $$ \big|\Lambda( x , y) \big| \leq1 \quad{\text{for all }} x, y \in \mathbb {R}^{d}. $$
  We denote by $L_{\beta}^{p}(\overline{\mathbb {R}^{d}_{+}})$, $1 \leq p \leq \infty$, the space of measurable functions on $\overline{\mathbb {R}^{d}_{+}}$ such that
  $$\begin{gathered} \|f\|_{L^{p}_{\beta}(\overline{\mathbb {R}^{d}_{+}})} = \biggl( \int_{\overline{\mathbb {R}^{d}_{+}}} \big|f(y)\big|^{p} \,d\lambda_{\beta}(y) \biggr)^{\frac{1}{p}} < \infty \quad{\text{if }} 1 \leq p < \infty, \\ \|f\|_{L^{\infty}_{\beta}(\overline{\mathbb {R}^{d}_{+}})} = {\operatorname {ess}} \sup_{y \in\overline{\mathbb {R}^{d}_{+}} } \big|f(y)\big| < \infty\quad{\text{if }} p = \infty, \end{gathered} $$
  where $d\lambda_{\beta}$ is the measure on $\overline{\mathbb {R}^{d}_{+}}$ given by
  $$d\lambda_{\beta}(y) := y_{d}^{2\beta+ 1}dy'\, dy_{d}. $$

The Weinstein transform is given for *f* in $L_{\beta}^{1}(\overline{\mathbb {R}^{d}_{+}})$ by

2.5 $$ \mathcal{F}_{W}(f) (\xi) = \frac{1}{C(\beta)} \int_{\mathbb {R}^{d+1}_{+}}f(y) \Lambda(-\xi,y) \,d\lambda_{\beta}(y) \quad{\text{for all }} \xi \in \overline{\mathbb {R}^{d}_{+}}, $$

where

$$C(\beta) = \int_{\overline{\mathbb {R}^{d}_{+}}}e^{-\frac{\|y\|^{2}}{2}}\, d\lambda_{\beta}(y)= 2^{\beta}\Gamma(\beta+1)(2\pi)^{\frac{d-1}{2}}. $$

Some basic properties of this transform are the following:

(i) For *f* in $L_{\beta}^{1}(\overline{\mathbb {R}^{d}_{+}})$,
  2.6 $$ \big\| \mathcal{F}_{W} (f)\big\| _{L^{\infty}_{\beta}(\overline{\mathbb {R}^{d}_{+}})} \leq\frac{1}{C(\beta)} \|f \|_{L^{1}_{\beta}(\overline{\mathbb {R}^{d}_{+}})}. $$
(ii) For all *f* in $L_{\beta}^{1}(\overline{\mathbb {R}^{d}_{+}})$, if $\mathcal {F}_{W}(f)$ belongs to $L_{\beta}^{1}(\overline{\mathbb {R}^{d}_{+}})$, then
  2.7 $$ f(y) = \frac{1}{C(\beta)} \int_{\overline{\mathbb {R}^{d}_{+}}} \mathcal {F}_{W} (f) ( \xi) \Lambda(\xi,y) \,d\lambda_{\beta}(\xi) \quad\mbox{a.e.} $$
(iii) For $f\in\mathcal{S}_{*}(\mathbb {R}^{d})$, if we define
  $$ \overline{\mathcal{F}_{W}}(f) (\xi)=\mathcal{F}_{W}(f) (- \xi), $$
  then
  2.8 $$ {\mathcal{F}_{W}}\overline{\mathcal{F}_{W}} = \overline{\mathcal{F}_{W}} {{\mathcal{F}}_{W}} = \mathit{Id}. $$

#### Proposition 2.1

(i) *The Weinstein transform* $\mathcal{F}_{W}$ *is a topological isomorphism from* $\mathcal{S}_{*}(\mathbb {R}^{d})$ *onto itself*, *and for all f in* $\mathcal{S}_{*}(\mathbb {R}^{d})$,
  2.9 $$ \int_{\overline{\mathbb {R}^{d}_{+}}} \big|f(y)\big|^{2} \,d \lambda_{\beta}(y) = \int_{\overline{\mathbb {R}^{d}_{+}}}\big|\mathcal {F}_{W}(f) ( \xi)\big|^{2} \,d \lambda_{\beta}(\xi). $$
(ii) *The Weinstein transform* $f \to\mathcal{F}_{W}(f)$ *induces an isometric isomorphism from* $L_{\beta}^{2}(\overline{\mathbb {R}^{d}_{+}})$ *onto itself*.

For a function $f\in\mathcal{S}_{*}(\mathbb {R}^{d})$ and $y\in\overline{\mathbb {R}^{d}_{+}}$, the generalized translation $\tau_{y} f$ is defined by the following relation:

2.10 $$ \forall\xi\in \mathbb {R}^{d}, \quad\mathcal{F}_{W} ( \tau_{y}f) (\xi)= \Lambda(\xi,y) \mathcal{F}_{W} (f) (\xi). $$

By using the generalized translation, we define the generalized convolution product $f*_{W}g$ of functions $f, g\in L^{1}_{\beta}(\overline{\mathbb {R}^{d}_{+}})$ as follows:

2.11 $$ f*_{W}g(x)= \int_{\overline{\mathbb {R}^{d}_{+}}}\tau_{x}f \bigl(-y',y_{d} \bigr)g(y)\, d\lambda_{\beta}(y), \quad x \in \overline{\mathbb {R}^{d}_{+}}. $$

This convolution is commutative and associative. Moreover, we have the following:

#### Proposition 2.2

(i) *For all* $f, g\in L^{1}_{\beta}(\overline{\mathbb {R}^{d}_{+}})$, $f*_{W}g$ *belongs to* $L^{1}_{\beta}(\overline{\mathbb {R}^{d}_{+}})$, *and*
  2.12 $$ \forall\xi\in\overline{\mathbb {R}^{d}_{+}}, \quad \mathcal{F}_{W} (f*_{W}g) (\xi) = \mathcal{F}_{W} (f) (\xi)\mathcal{F}_{W} (g) (\xi). $$
(ii) *Let* $1\leq p, q, r\leq\infty$ *be such that* $\frac{1}{p}+\frac{1}{q}-\frac{1}{r}=1$. *If* $f\in L^{p}_{\beta}(\overline{\mathbb {R}^{d}_{+}})$ *and* $g\in L^{q}_{\beta }(\overline{\mathbb {R}^{d}_{+}})$, *then* $f*_{W}g\in L^{r}_{\beta}(\overline{\mathbb {R}^{d}_{+}})$, *and*
  2.13 $$ \Vert f*_{W}g \Vert _{L^{r}_{\beta}(\overline{\mathbb {R}^{d}_{+}})} \leq \Vert f \Vert _{L^{p}_{\beta}(\overline{\mathbb {R}^{d}_{+}})} \Vert g \Vert _{L^{q}_{\beta}(\overline{\mathbb {R}^{d}_{+}})}. $$
(iii) *Let* *f*, *g* *be in* $L^{2}_{\beta}(\overline{\mathbb {R}^{d}_{+}})$. *Then* $f*_{W}g \in L^{2}_{\beta}(\overline{\mathbb {R}^{d}_{+}})$ *if and only if* ${\mathcal {F}}_{W} (f){\mathcal {F}}_{W} (g) \in L^{2}_{\beta}(\overline{\mathbb {R}^{d}_{+}})$ *and* (2.12) *holds*.

#### Definition 2.1

Let *E*, *F* be two measurable subsets of $\overline{\mathbb {R}^{d}_{+}}$. Then:

1. We say that $(E,F)$ is weakly annihilating pair if $\operatorname{supp} f \subset E$ and $\operatorname{supp} \mathcal{F}_{W}(f) \subset F$ implies $f = 0$.
2. We say that $(E,F)$ is strongly annihilating pair if there exists a constant $C:=C_{\beta}(E,F)>0$ such that, for every function $f \in L^{2}_{\beta}(\overline{\mathbb {R}^{d}_{+}})$,
  2.14 $$ C \bigl(\big\| \mathcal{F}_{W}(f)\big\| ^{2}_{L^{2}_{\beta}(F^{c})}+ \|f \|_{L^{2}_{\beta }(E^{c})}^{2} \bigr) \geq\|f\|_{L^{2}_{\beta}(\overline{\mathbb {R}^{d}_{+}})}^{2}. $$

The constant $C_{ \beta} (E,F)$ is called the annihilation constant of $(E,F)$.

#### Proposition 2.3

[18] *Let* *E*, *F* *be two measurable subsets of* $\overline{\mathbb {R}^{d}_{+}}$ *with*

$$\int_{E}d\lambda_{\beta}(y) < \infty\quad{\textit{and}} \quad \int_{F}d\lambda_{\beta}(\xi) < \infty. $$

*Then the pair* $(E,F)$ *is a strongly annihilating pair*.

### Weinstein wavelets

This subsection gives an introduction to the theory of Weinstein wavelets. The main reference is [13].

#### Definition 2.2

A Weinstein wavelet on $\overline{\mathbb {R}^{d}_{+}}$ is a measurable function *h* on $\overline{\mathbb {R}^{d}_{+}}$ satisfying, for almost all $\xi\in\overline{\mathbb {R}^{d}_{+}}$, the condition

2.15 $$ 0 < \mathcal{C}_{h} = \int_{0}^{\infty}\big| \mathcal{F}_{W}(h) (t \xi)\big|^{2}\frac{dt}{t} < \infty. $$

#### Example 2.1

Let $h \in L^{2}_{\beta}(\mathbb {R}^{d}_{+})$ be given by

2.16 $$ h := \mathcal{F}_{W}^{-1} \bigl(\|y \|^{2}e^{-\frac{1}{2}\|y\|^{2}} \bigr). $$

By a simple calculations we see that, for almost all $x\in \mathbb {R}^{d}_{+}$,

$$\int_{0}^{\infty}\big|\mathcal{F}_{W}(h) (\lambda x)\big|^{2}\frac{d\lambda}{\lambda} < \infty. $$

Thus *h* is an example of a Weinstein wavelet on $\mathbb {R}^{d}_{+}$.

Let $b > 0$, and let *h* be in $L^{2}_{\beta}(\overline{\mathbb {R}^{d}_{+}})$. The dilation of *h* by *b* is defined by

2.17 $$ \forall y \in\overline{\mathbb {R}^{d}_{+}}, \quad h_{b}(y) = \frac{1}{b^{ 2\beta+1+ d}}h \biggl(\frac{y}{b} \biggr). $$

It is easy to see that $h_{b} \in L^{2}_{\beta}(\overline{\mathbb {R}^{d}_{+}})$ and

2.18 $$ \forall\xi\in\overline{\mathbb {R}^{d}_{+}}, \quad \mathcal{F}_{W}(h_{b}) (\xi) = \mathcal{F}_{W}(h) ({b\xi}). $$

We introduce the family $h_{b,y}$, $b > 0$ and $y \in\overline{\mathbb {R}^{d}_{+}}$, of Weinstein wavelets on $\overline{\mathbb {R}^{d}_{+}}$ in $L^{2}_{\beta}(\overline{\mathbb {R}^{d}_{+}})$ defined by

2.19 $$ \forall x \in\overline{\mathbb {R}^{d}_{+}}, \quad h_{b,y}(x) = b^{\beta +\frac{d+1}{2}} \tau_{x}h_{b} \bigl(-y',y_{d} \bigr). $$

Note that

2.20 $$ \forall b > 0, \forall y \in\overline{\mathbb {R}^{d}_{+}}, \quad\|h_{b,y}\|_{L^{2}_{\beta}(\overline{\mathbb {R}^{d}_{+}})} \leq\|h\| _{L^{2}_{\beta}(\overline{\mathbb {R}^{d}_{+}})}. $$

#### Notation

We denote:

$$\Omega_{d+1} = \bigl\{ (b,y)=(b,y_{1},\ldots,y_{d}) \in \mathbb {R}^{d+1}, b > 0 {\text{ and }} y \in\overline{\mathbb {R}^{d}_{+}} \bigr\} . $$

$L^{p}_{\mu_{\beta}}(\Omega_{d+1})$, $1\leq p \leq\infty$, is the space of measurable functions *f* on $\Omega_{d+1}$ such that

$$\begin{gathered} {\|f\|} _{L^{p}_{\mu_{\beta}}(\Omega_{d+1})} :=\biggl( \int _{\Omega_{d+1}}\big|f(b,y)\big|^{p}d{ \mu}_{\beta} (b,y) \biggr)^{\frac{1}{p}}< \infty, \quad1\leq p< \infty, \\ {\|f\|} _{L^{\infty}_{\mu_{\beta}}(\Omega_{d+1})} := \operatorname{ess}\sup _{(b,y)\in\Omega_{d+1}} {\big|f(b,y)\big|< \infty, \quad p = \infty,} \end{gathered}$$

where the measure $d\mu_{\beta}$ is defined by

$$\forall(b,y) \in\Omega_{d+1}, \quad d{\mu}_{\beta}(b,y)= \frac{y_{d}^{2\beta+1}\,dy\,db}{b^{2\beta+d+2}}. $$

#### Definition 2.3

Let *h* be a Weinstein wavelet on $\overline{\mathbb {R}^{d}_{+}}$ in $L^{2}_{\beta}(\overline{\mathbb {R}^{d}_{+}})$. The Weinstein continuous wavelet transform $\mathcal{S}_{h}^{W}$ on $\overline{\mathbb {R}^{d}_{+}}$ is defined for regular functions *f* on $\overline{\mathbb {R}^{d}_{+}}$ by

2.21 $$ \mathcal{S}_{h}^{W}(f) (b,y) = \int_{\overline{\mathbb {R}^{d}_{+}}}f(y)\overline{h_{b,y}(x)} \,d \lambda_{\beta}(x), \quad(b,y) \in{\Omega_{d+1}}. $$

It is easy to see that

2.22 $$ \forall(b,y) \in{\Omega_{d+1}}, \quad \mathcal{S}_{h}^{W}(f) (b,y) = b^{\beta+ \frac{d+1}{2}}f *_{W}\overline{h_{b}}(y). $$

#### Proposition 2.4

*Let* *f* *be in* $L^{2}_{\beta}(\overline{\mathbb {R}^{d}_{+}})$, *and let* *h* *be a Weinstein wavelet*. *Then we have*

2.23 $$ \mathcal{S}_{h}^{W}(f) (b,y) = b^{\beta+ \frac{d+1}{2}}\mathcal{F}_{W}^{-1} \bigl( \mathcal{F}_{W}(f) (\xi) \mathcal{F}_{W}(\overline{h}) (b\xi) \bigr) (y), \quad(b,y) \in\Omega_{d+1}, $$

*and*

2.24 $$ {\big\| \mathcal{S}_{h}^{W}f\big\| } _{L^{\infty }_{\mu_{\beta}}(\Omega_{d+1})} \leq{\| f\|}_{L^{2}_{\beta}(\overline{\mathbb {R}^{d}_{+}})} {\| h\| }_{L^{2}_{\beta}(\overline{\mathbb {R}^{d}_{+}})}. $$

#### Proposition 2.5

(Covariance properties)

*Let* *h* *be a Weinstein wavelet*. *The transformation* $\mathcal{S}_{h}^{W}$ *is a bounded linear operator from* $L^{2}_{\beta}(\overline{\mathbb {R}^{d}_{+}})$ *into the space of continuous bounded functions on* $\Omega_{d+1}$. *Moreover*, *we have the following covariance property*:

*For any* $f \in L^{2}_{\beta}(\overline{\mathbb {R}^{d}_{+}})$, $(b,y) \in\Omega_{d+1}$ *and* $c \in \mathbb {R}^{*}$,

2.25 $$ \biggl[\mathcal{S}_{h}^{W} \biggl( \frac{1}{ c^{\beta+ \frac{d}{2}}}f \biggl(\frac{.}{c} \biggr) \biggr) \biggr](b,y) = \bigl[ \mathcal{S}_{h}^{W}(f) \bigr] \biggl( \frac{b}{c}, \frac{y}{c} \biggr). $$

#### Theorem 2.1

(Plancherel’s formula for $\mathcal{S}_{h}^{W}$)

*Let* *h* *be a Weinstein wavelet*. *For all* *f* *in* $L^{2}_{\beta}(\overline{\mathbb {R}^{d}_{+}})$, *we have*

2.26 $$ \int_{\overline{\mathbb {R}^{d}_{+}}}\big|f(x)\big|^{2}\,d\lambda_{\beta}(x) = \frac{1}{\mathcal{C}_{h}} \int_{0}^{\infty} \int_{\overline{\mathbb {R}^{d}_{+}}} \big| \mathcal{S}_{h}^{W}(f) (b,y)\big|^{2} \,d{ \mu}_{\beta}(b,y). $$

#### Corollary 2.1

(Parseval’s formula)

*Let* *h* *be a Weinstein wavelet*. *For all* $f_{1}$, $f_{2}$ *in* $L^{2}_{\beta}(\overline{\mathbb {R}^{d}_{+}})$, *we have*

2.27 $$ \int_{\overline{\mathbb {R}^{d}_{+}}}f_{1}(x)\overline{f_{2}(x)} \,d \lambda_{\beta}(x) = \frac{1}{\mathcal{C}_{h}} \int_{0}^{\infty} \int_{\overline{\mathbb {R}^{d}_{+}}} \mathcal{S}_{h}^{W}(f_{1}) (b,y)\overline{\mathcal{S}_{h}^{W}(f_{2}) (b,y)} \,d{\mu}_{\beta}(b,y). $$

## Localization operators for the Weinstein continuous wavelet transform

### Preliminaries

We denote by $B(L^{2}_{\beta}(\overline{\mathbb {R}^{d}_{+}}))$ the space of bounded operators from $L^{2}_{\beta}(\overline{\mathbb {R}^{d}_{+}})$ into $L^{2}_{\beta}(\overline{\mathbb {R}^{d}_{+}})$. The singular values $(s_{n}(M))_{n\in \mathbb {N}}$ of a compact operator $M \in B(L^{2}_{\beta}(\overline{\mathbb {R}^{d}_{+}}))$ are the eigenvalues of the positive self-adjoint operator $|M| = \sqrt{M^{*}M}$. For $1 \leq p < \infty$, the Schatten class $S_{p}$ is the space of compact operators whose singular values lie in $l^{p}$. Hence $S_{p}$ is equipped with the norm

3.1 $$ \|M\|_{S_{p}}:= \Biggl(\sum_{j=1}^{\infty} \bigl(s_{j}(M) \bigr)^{p} \Biggr)^{\frac{1}{p}}. $$

In particular, $S_{2}$ is the space of Hilbert-Schmidt operators, and $S_{1}$ is the space of trace-class operators. It is well known that the trace of an operator $M \in S_{1}$ is defined by

3.2 $$ \operatorname{tr}(M) = \sum_{j=1}^{\infty} \langle M v_{j},v_{j}\rangle, $$

where $(v_{j})_{j}$ is any orthonormal basis for $L^{2}_{\beta}(\overline {\mathbb {R}^{d}_{+}})$. Moreover, if *M* is positive, then

3.3 $$ \operatorname{tr}(M) = \|M\|_{S_{1}}. $$

Moreover, a compact operator *M* on the Hilbert space $L^{2}_{\beta }(\overline{\mathbb {R}^{d}_{+}})$ is Hilbert-Schmidt if the positive operator $M^{*}M$ is in the space of trace class $S_{1}$. Then

3.4 $$ \|M\|_{HS}^{2}:= \|M\|_{S_{2}}^{2} = \big\| M^{*}M\big\| _{S_{1}} = \operatorname{tr} \bigl(M^{*}M \bigr) = \sum_{j=1}^{\infty}\|M v_{j} \|_{L^{2}_{\beta}(\overline{\mathbb {R}^{d}_{+}})}^{2} $$

for any orthonormal basis $(v_{j})_{j}$ basis for $L^{2}_{\beta}(\overline {\mathbb {R}^{d}_{+}})$.

For consistency, we define $S_{\infty}:= B(L^{2}_{\beta}(\overline {\mathbb {R}^{d}_{+}}))$ equipped with norm

3.5 $$ \|M\|_{S_{\infty}}:= \sup_{v \in L^{2}_{\beta }(\overline{\mathbb {R}^{d}_{+}}): \|v\|_{L^{2}_{\beta}(\overline{\mathbb {R}^{d}_{+}})} = 1}\|M v \|_{L^{2}_{\beta}(\overline{\mathbb {R}^{d}_{+}})}. $$

### Boundedness

In this section, *h* and *k* will be two Weinstein wavelets such that

$$\|h\|_{L^{2}_{\beta}(\overline{\mathbb {R}^{d}_{+}})}= \|k\|_{L^{2}_{\beta }(\overline{\mathbb {R}^{d}_{+}})}=1. $$

#### Definition 3.1

The localization operator associated with the symbol *a* and two Weinstein wavelets is denoted by $\mathcal {L}_{h,k}(a)$ and defined on $L^{2}_{\beta}(\overline{\mathbb {R}^{d}_{+}})$ by

$$\mathcal {L}_{h,k}(a)(f) (x)= \frac{1}{\sqrt{\mathcal{C}_{h}\mathcal{C}_{k}}} \int _{\Omega_{d+1}}a(b,y) \mathcal{S}_{h}^{W}(f) (b,y) k_{b,y}(x)\,d\mu_{\beta}(b,y),\quad x \in\overline{ \mathbb {R}^{d}_{+}}. $$

Often, it is more convenient to interpret the definition of $\mathcal {L}_{h,k}(a)$ in a weak sense, that is, for *f*, *g* in $L^{2}_{\beta}(\overline{\mathbb {R}^{d}_{+}})$,

3.6 $$ \begin{aligned}[b]&\bigl\langle \mathcal {L}_{h,k}(a)(f), g \bigr\rangle _{L^{2}_{\beta }(\overline{\mathbb {R}^{d}_{+}})}\\&\quad= \frac{1}{\sqrt{\mathcal{C}_{h}\mathcal{C}_{k}}} \int_{\Omega _{d+1}} a(b,y)\mathcal{S}_{h}^{W}(f) (b,y)\overline{\mathcal{S}_{k}^{W}(g) (b,y)}\,d \mu_{\beta}(b,y),\quad g\in L^{2}_{\beta } \bigl(\overline{ \mathbb {R}^{d}_{+}} \bigr).\end{aligned} $$

#### Proposition 3.1

*The adjoint of* $\mathcal {L}_{h,k}(a)$ *is* ${\mathcal {L}_{k,h}(\overline{a})}$.

#### Proof

For all *f*, *g* in $L^{2}_{\beta}(\overline{\mathbb {R}^{d}_{+}})$, from (3.6) it immediately follows that

$$\begin{aligned} \bigl\langle \mathcal {L}_{h,k}(a)(f), g \bigr\rangle _{L^{2}_{\beta }(\overline{\mathbb {R}^{d}_{+}})}&= \frac{1}{\sqrt{\mathcal{C}_{h}\mathcal{C}_{k}}} \int_{\Omega _{d+1}} a(b,y)\mathcal{S}_{h}^{W}(f) (b,y)\overline{\mathcal{S}_{k}^{W}(g) (b,y)}\,d{ \mu}_{\beta}(b,y) \\ &= \overline{\frac {1}{\sqrt{\mathcal{C}_{h}\mathcal{C}_{k}}} \int_{\Omega_{d+1}} \overline{a(b,y)} \mathcal{S}_{k}^{W}(g) (b,y)\overline{\mathcal {S}_{h}^{W}(f) (b,y)}\,d{ \mu}_{\beta}(b,y)} \\ &= \overline{ \bigl\langle {\mathcal{L}_{k,h}(\overline{a})}(g), f \bigr\rangle _{L^{2}_{\beta }(\overline{\mathbb {R}^{d}_{+}})}} = \bigl\langle f, {\mathcal{L}_{k,h}( \overline{a})}(g) \bigr\rangle _{L^{2}_{\beta}(\overline{\mathbb {R}^{d}_{+}})}. \end{aligned} $$

Thus we get

3.7 $$ {\mathcal{L}^{*}_{h,k}(a)} = {\mathcal {L}_{k,h}(\overline{a})}. $$

 □

The objective of this subsection is to prove that the operators

$$\mathcal {L}_{h,k}(a):L^{2}_{\beta} \bigl(\overline{\mathbb {R}^{d}_{+}} \bigr)\rightarrow L^{2}_{\beta } \bigl(\overline{ \mathbb {R}^{d}_{+}} \bigr) $$

are bounded for all symbols $a \in L^{p}_{\mu_{\beta}}(\Omega _{d+1})$. We first consider this problem for *a* in $L^{1}_{\mu_{\beta }}(\Omega_{d+1})$ and next in $L^{\infty}_{\mu_{\beta}}(\Omega_{d+1})$, and then by interpolation theory we deduce the result.

#### Proposition 3.2

*Let* *a* *be in* $L^{1}_{\mu_{\beta}}(\Omega_{d+1})$. *Then the localization operator* $\mathcal {L}_{h,k}(a)$ *is in* $S_{\infty}$, *and*

$$\big\| \mathcal {L}_{h,k}(a)\big\| _{S_{\infty}} \leqslant\frac{1}{\sqrt{\mathcal {C}_{h}\mathcal{C}_{k}}} \|a\|_{L^{1}_{\mu_{\beta}}(\Omega_{d+1})}. $$

#### Proof

Let *f* and *g* be in $L^{2}_{\beta}(\overline{\mathbb {R}^{d}_{+}})$. From relations (3.6) and (2.24) we have

$$\begin{aligned} \big| \bigl\langle \mathcal {L}_{h,k}(a)(f), g \bigr\rangle _{L^{2}_{\beta}(\overline {\mathbb {R}^{d}_{+}})}\big|&\leqslant \frac{1}{\sqrt{\mathcal{C}_{h}\mathcal {C}_{k}}} \int_{\Omega_{d+1}} \big| a(b,y)\big|\big|\mathcal{S}_{h}^{W}(f) (b,y)\big|\big|\overline{\mathcal{S}_{k}^{W}(g) (b,y)}\big|\,d\mu _{\beta}(b,y) \\ &\leqslant\frac{1}{\sqrt{\mathcal{C}_{h}\mathcal {C}_{k}}}\big\| \mathcal{S}_{h}^{W}(f) \big\| _{L^{\infty}_{\mu_{\beta }}(\Omega_{d+1})}\big\| \mathcal{S}_{k}^{W}(g)\big\| _{L^{\infty}_{\mu_{\beta }}(\Omega_{d+1})} \|a\|_{L^{1}_{\mu_{\beta}}(\Omega_{d+1})} \\ &\leqslant\frac{1}{\sqrt{\mathcal{C}_{h}\mathcal{C}_{k}}}\|f\| _{L^{2}_{\beta }(\overline{\mathbb {R}^{d}_{+}})}\|g\|_{L^{2}_{\beta}(\overline{\mathbb {R}^{d}_{+}})}\|a \|_{L^{1}_{\mu_{\beta}}(\Omega_{d+1})}.\end{aligned} $$

Thus,

$$\big\| \mathcal {L}_{h,k}(a)\big\| _{S_{\infty}} \leqslant\frac{1}{\sqrt{\mathcal {C}_{h}\mathcal{C}_{k}}} \|a\|_{L^{1}_{\mu_{\beta}}(\Omega_{d+1})}. $$

 □

#### Proposition 3.3

*Let* *a* *be in* $L^{\infty}_{\mu_{\beta}}(\Omega_{d+1})$. *Then the localization operator* $\mathcal {L}_{h,k}(a)$ *is in* $S_{\infty}$, *and*

$$\big\| \mathcal {L}_{h,k}(a)\big\| _{S_{\infty}}\leqslant\|a\|_{L^{\infty}_{\mu_{\beta }}(\Omega_{d+1})}. $$

#### Proof

For all functions *f* and *g* in $L^{2}_{\beta}(\overline{\mathbb {R}^{d}_{+}})$, by Hölder’s inequality we have

$$\begin{aligned} \big| \bigl\langle \mathcal {L}_{h,k}(a)(f), g \bigr\rangle _{L^{2}_{\beta}(\overline {\mathbb {R}^{d}_{+}})}\big|&\leqslant \frac{1}{\sqrt{\mathcal{C}_{h}\mathcal {C}_{k}}} \int_{\Omega_{d+1}} \big| a(b,y)\big|\big|\mathcal{S}_{h}^{W}(f) (b,y)\big|\big|\overline{\mathcal{S}_{k}^{W}(g) (b,y)}\big|\,d\mu _{\beta}(b,y) \\ &\leqslant\frac{1}{\sqrt{\mathcal{C}_{h}\mathcal {C}_{k}}}\|a\|_{L^{\infty}_{\mu_{\beta}}(\Omega_{d+1})}\big\| \mathcal {S}_{h}^{W}(f) \big\| _{L^{2}_{\mu_{\beta}}(\Omega_{d+1})}\big\| \mathcal{S}_{k}^{W}(g) \big\| _{L^{2}_{\mu_{\beta}}(\Omega_{d+1})}.\end{aligned} $$

Applying the Plancherel formula (2.26) for $\mathcal{S}_{h}^{W}$ and $\mathcal{S}_{k}^{W}$, we obtain

$$ \big| \bigl\langle \mathcal {L}_{h,k}(a)(f), g \bigr\rangle _{L^{2}_{\beta}(\overline {\mathbb {R}^{d}_{+}})}\big|\leqslant\|a \|_{L^{\infty}_{\mu_{\beta}}(\Omega _{d+1})} \|f\|_{L^{2}_{\beta}(\overline{\mathbb {R}^{d}_{+}})}\|g\| _{L^{2}_{\beta}(\overline{\mathbb {R}^{d}_{+}})}. $$

Thus,

$$\big\| \mathcal {L}_{h,k}(a)\big\| _{S_{\infty}}\leqslant\|a\|_{L^{\infty}_{\mu_{\beta }}(\Omega_{d+1})}. $$

 □

#### Remark 3.1

For $a=a_{1}+a_{\infty}$ in $L^{1}_{\mu_{\beta}}(\Omega _{d+1})+L^{\infty}_{\mu_{\beta}}(\Omega_{d+1})$, from Proposition 3.2 and Proposition 3.3 we deduce that $\mathcal {L}_{h,k}(a)$ is in $S_{\infty}$ and

$$\big\| \mathcal {L}_{h,k}(a)\big\| _{S_{\infty}} \leqslant\frac{1}{\sqrt{\mathcal {C}_{h}\mathcal{C}_{k}}}\|a_{1} \|_{L^{1}_{\mu_{\beta}}(\Omega _{d+1})}+ \|a_{\infty}\|_{L^{\infty}_{\mu_{\beta}}(\Omega _{d+1})}\leqslant\max \biggl\{ \frac{1}{\sqrt{\mathcal{C}_{h}\mathcal {C}_{k}}},1 \biggr\} \|a\|, $$

where

$$\|a\|=\inf \bigl\{ \|a_{1}\|_{L^{1}_{\mu_{\beta}}(\Omega_{d+1})}+ \| a_{\infty}\|_{L^{\infty}_{\mu_{\beta}}(\Omega_{d+1})}: a=a_{1}+a_{\infty}, a_{1}\in L^{1}_{\mu_{\beta}}(\Omega_{d+1}), a_{\infty}\in L^{\infty}_{\mu_{\beta}}(\Omega_{d+1}) \bigr\} . $$

We now can prove that $\mathcal {L}_{h,k}(a)$ is in $S_{\infty}$ for every symbol *a* in $L^{p}_{\mu_{\beta}}(\Omega_{d+1})$, $1 \leq p \leq\infty$.

#### Theorem 3.1

*Let* *a* *be in* $L^{p}_{\mu_{\beta}}(\Omega _{d+1})$, $p\in[1,\infty]$. *Then there exists a bounded linear operator* $\mathcal {L}_{h,k}(a):L^{2}_{\beta}(\overline{\mathbb {R}^{d}_{+}})\rightarrow L^{2}_{\beta}(\overline{\mathbb {R}^{d}_{+}})$ *such that*

$$\big\| \mathcal {L}_{h,k}(a)\big\| _{S_{\infty}}\leqslant \biggl(\frac{1}{\mathcal {C}_{h}\mathcal {C}_{k}} \biggr)^{\frac{1}{2p}} \|a\|_{L^{p}_{\mu_{\beta}}(\Omega_{d+1})}. $$

#### Proof

Let *f* be in $L^{2}_{\beta}(\overline{\mathbb {R}^{d}_{+}})$. We consider the operator

$$\mathcal{T}: L^{1}_{\mu_{\beta}}( \Omega_{d+1}) \cap L^{\infty }_{\mu_{\beta}}( \Omega_{d+1}) \rightarrow L^{2}_{\beta} \bigl( \mathbb {R}^{d} \bigr) $$

given by

$$\mathcal{T}(a):= \mathcal {L}_{h,k}(a)(f). $$

Then, by Proposition 3.2 and Proposition 3.3,

3.8 $$ \big\| \mathcal{T}(a)\big\| _{L^{2}_{\beta}(\overline{\mathbb {R}^{d}_{+}})} \leq\frac{1}{\sqrt{\mathcal{C}_{h}\mathcal{C}_{k}}}\|f \|_{L^{2}_{\beta }(\overline{\mathbb {R}^{d}_{+}})} \|a\|_{L^{1}_{\mu_{\beta}}(\Omega_{d+1})} $$

and

3.9 $$ \big\| \mathcal{T}(a)\big\| _{L^{2}_{\beta}(\overline{\mathbb {R}^{d}_{+}})} \leq\|f\|_{L^{2}_{\beta}(\overline{\mathbb {R}^{d}_{+}})} \|a \|_{L^{\infty}_{\mu _{\beta}}(\Omega_{d+1})}. $$

Therefore, by (3.8), (3.9), and the Riesz-Thorin interpolation argument ([19, Theorem 2], see also [17, Theorem 2.11]) we get that $\mathcal{T}$ may be uniquely extended to a linear operator on $L^{p}_{\mu_{\beta}}(\Omega _{d+1})$, $1 \leq p \leq\infty$, and we have

3.10 $$ \big\| \mathcal {L}_{h,k}(a)(f)\big\| _{L^{2}_{\beta}(\overline{\mathbb {R}^{d}_{+}})} = \big\| \mathcal{T}(a)\big\| _{L^{2}_{\beta}(\overline{\mathbb {R}^{d}_{+}})} \leq \biggl(\frac {1}{\sqrt{\mathcal{C}_{h}\mathcal{C}_{k}}} \biggr)^{\frac {1}{p}}\|f\|_{L^{2}_{\beta}(\overline{\mathbb {R}^{d}_{+}})} \|a\| _{L^{p}_{\mu_{\beta}}(\Omega_{d+1})}. $$

Since (3.10) is true for all $f \in L^{2}_{\beta }(\overline{\mathbb {R}^{d}_{+}})$, the result is proved. □

### Schatten-von Neumann properties for $\mathcal {L}_{h,k}(a)$

Let us begin with the following statement.

#### Proposition 3.4

*Let* *a* *be in* $L^{1}_{\mu_{\beta}}(\Omega_{d+1})$. *Then the localization operator*

$$\mathcal {L}_{h,k}(a): L^{2}_{\beta} \bigl(\overline{\mathbb {R}^{d}_{+}} \bigr)\rightarrow L^{2}_{\beta } \bigl(\overline{ \mathbb {R}^{d}_{+}} \bigr) $$

*is in* $S_{2}$, *and*

$$\big\| \mathcal {L}_{h,k}(a)\big\| _{S_{2}}\leqslant\frac{1}{\sqrt{\mathcal{C}_{h}\mathcal {C}_{k}}}\|a\|_{L^{1}_{\mu_{\beta}}(\Omega_{d+1})}. $$

#### Proof

Let $\{\phi_{j}, j=1,2,\ldots\}$ be an orthonormal basis for $L^{2}_{\beta }(\overline{\mathbb {R}^{d}_{+}})$. Then by (3.6), Fubini’s theorem, Parseval’s identity, and relations (2.21) and (3.7) we have

$$\begin{gathered} \sum_{j=1}^{\infty} \big\| \mathcal {L}_{h,k}(a)(\phi_{j})\big\| _{L^{2}_{\beta }(\overline{\mathbb {R}^{d}_{+}}))}^{2} \\\quad= \sum _{j=1}^{\infty} \bigl\langle \mathcal {L}_{h,k}(a)(\phi_{j}), \mathcal {L}_{h,k}(a)(\phi_{j}) \bigr\rangle _{L^{2}_{\beta}(\overline{\mathbb {R}^{d}_{+}})} \\ \quad= \sum_{j=1}^{\infty} \frac{1}{\sqrt{\mathcal{C}_{h}\mathcal{C}_{k}}} \int_{\Omega_{d+1}} a(b,y)\langle\phi_{j}, h_{b,y} \rangle_{L^{2}_{\beta}(\overline{\mathbb {R}^{d}_{+}})}\overline{ \bigl\langle \mathcal {L}_{h,k}(a)(\phi_{j}), k_{b,y} \bigr\rangle _{L^{2}_{\beta}(\overline{\mathbb {R}^{d}_{+}})}} \, d{\mu}_{\beta}(b,y) \\ \quad= \frac{1}{\sqrt{\mathcal{C}_{h}\mathcal{C}_{k}}} \int_{\Omega _{d+1}} a(b,y)\sum_{j=1}^{\infty} \langle\phi_{j}, h_{{b,y}}\rangle_{L^{2}_{\beta}(\overline{\mathbb {R}^{d}_{+}})}{ \bigl\langle { \mathcal {L}^{*}_{h,k}(a)}( k_{{b,y}}), \phi_{j} \bigr\rangle _{L^{2}_{\beta }(\overline{\mathbb {R}^{d}_{+}})}} \,d{\mu}_{\beta}(b,y) \\ \quad=\frac{1}{\sqrt{\mathcal{C}_{h}\mathcal{C}_{k}}} \int_{\Omega _{d+1}} a(b,y) \bigl\langle { \mathcal{L}^{*}_{h,k}(a)} k_{{b,y}},h_{{b,y}} \bigr\rangle _{L^{2}_{\beta}(\overline{\mathbb {R}^{d}_{+}})} \,d{\mu }_{\beta}(b,y). \end{gathered} $$

Thus, we get

3.11 $$ \begin{aligned}[b]\sum_{j=1}^{\infty}\big\| \mathcal {L}_{h,k}(a)( \phi_{j})\big\| _{L^{2}_{\beta}(\overline{\mathbb {R}^{d}_{+}})}^{2} &\leq \frac{1}{\sqrt{\mathcal{C}_{h}\mathcal {C}_{k}}} \int_{\Omega_{d+1}} \big|a(b,y)\big| \big\| {\mathcal{L}^{*}_{h,k}(a)} \big\| _{S_{\infty}}\,d{\mu}_{\beta}(b,y) \\ &= \frac{1}{\sqrt{\mathcal{C}_{h}\mathcal{C}_{k}}} \big\| {\mathcal {L}^{*}_{h,k}(a)} \big\| _{S_{\infty}}\|a\|_{L^{1}_{\mu_{\beta}}(\Omega _{d+1})} < \infty.\end{aligned} $$

So by (3.11) and Proposition 2.8 in Wong [17]

$$\mathcal {L}_{h,k}(a): L^{2}_{\beta} \bigl(\overline{\mathbb {R}^{d}_{+}} \bigr)\rightarrow L^{2}_{\beta } \bigl(\overline{ \mathbb {R}^{d}_{+}} \bigr) $$

is in the Hilbert-Schmidt class $S_{2}$ and hence compact. □

#### Proposition 3.5

*Let* *a* *be a symbol in* $L^{p}_{\mu_{\beta}}(\Omega_{d+1})$, $p\in [1,\infty)$. *Then the operator* $\mathcal {L}_{h,k}(a)$ *is compact*.

#### Proof

Let *a* be in $L^{p}_{\mu_{\beta}}(\Omega_{d+1})$, and let a sequence $(a_{n})_{n\in \mathbb {N}} \in L^{1}_{\mu_{\beta}}(\Omega_{d+1}) \cap L^{\infty}_{\mu_{\beta}}(\Omega_{d+1})$ be such that $a_{n} \rightarrow a$ in $L^{p}_{\mu_{\beta}}(\Omega_{d+1})$ as $n \to\infty$. Then by Theorem 3.1

$$\big\| {\mathcal{L}_{h,k}(a_{n})} - \mathcal {L}_{h,k}(a)\big\| _{S_{\infty}} \leq \biggl(\frac{1}{\sqrt{\mathcal{C}_{h}\mathcal{C}_{k}}} \biggr)^{\frac{1}{p}}\| a_{n} - a \|_{L^{p}_{\mu_{\beta}}(\Omega_{d+1})}. $$

Hence ${\mathcal{L}_{h,k}(a_{n})} \to \mathcal {L}_{h,k}(a)$ in $S_{\infty}$ as $n \to\infty$. On the other hand, since by Proposition 3.4 ${\mathcal{L}_{h,k}(a_{n})} $ is in $S_{2}$ and hence compact, it follows that $\mathcal {L}_{h,k}(a)$ is compact. □

#### Theorem 3.2

*Let* *a* *be in* $L^{1}_{\mu _{\beta}}(\Omega_{d+1})$. *Then*

3.12 $$ \frac{2}{\mathcal{C}_{h}+\mathcal{C}_{k}}\| \widetilde{a}\| _{L^{1}_{\mu_{\beta}}(\Omega_{d+1})}\leqslant\big\| \mathcal {L}_{h,k}(a)\big\| _{S_{1}}\leqslant\frac{1}{\sqrt{\mathcal{C}_{h}\mathcal {C}_{k}}}\|a\|_{L^{1}_{\mu _{\beta}}(\Omega_{d+1})}, $$

*where* *ã* *is given by*

$$\widetilde{a}(b,y)= \bigl\langle \mathcal {L}_{h,k}(a)( h_{b,y}), k_{b,y} \bigr\rangle _{L^{2}_{\beta}(\overline{\mathbb {R}^{d}_{+}})}, \quad(b,y)\in \Omega_{d+1}. $$

#### Proof

Since *a* is in $L^{1}_{\mu_{\beta}}(\Omega_{d+1})$, $\mathcal {L}_{h,k}(a)$ is in $S_{2}$ by Proposition 3.4. By [17, Theorem 2.2] there exist an orthonormal basis $\{\phi_{j}, j=1,2,\ldots\}$ for the orthogonal complement of the kernel of $\mathcal {L}_{h,k}(a)$, consisting of eigenvectors of $|\mathcal {L}_{h,k}(a)|$, and an orthonormal set $\{\varphi_{j}, j=1,2,\ldots\}$ in $L^{2}_{\beta}(\overline{\mathbb {R}^{d}_{+}})$ such that

3.13 $$ \mathcal {L}_{h,k}(a)(f)= \sum_{j=1}^{\infty} s_{j}\langle f,\phi_{j}\rangle_{L^{2}_{\beta}(\overline{\mathbb {R}^{d}_{+}})} \varphi_{j}, $$

where $s_{j}, j\in \mathbb {N}$, are the positive singular values of $\mathcal {L}_{h,k}(a)$ corresponding to $\phi_{j}$. Thus, we get

$$ \big\| \mathcal {L}_{h,k}(a)\big\| _{S_{1}}= \sum_{j=1}^{\infty} s_{j}= \sum_{j=1}^{\infty} \bigl\langle \mathcal {L}_{h,k}(a)(\phi_{j}),\varphi_{j} \bigr\rangle _{L^{2}_{\beta }(\overline {\mathbb {R}^{d}_{+}})}. $$

Hence, by Fubini’s theorem, Cauchy-Schwarz’s inequality, Bessel inequality, and relations (2.21) and (2.20) we obtain

$$\begin{aligned} \big\| \mathcal {L}_{h,k}(a)\big\| _{S_{1}}={}& \sum_{j=1}^{\infty} \bigl\langle \mathcal {L}_{h,k}(a)(\phi_{j}), \varphi_{j} \bigr\rangle _{L^{2}_{\beta}(\overline{\mathbb {R}^{d}_{+}})} \\ ={}& \sum_{j=1}^{\infty} \frac{1}{\sqrt{\mathcal{C}_{h}\mathcal{C}_{k}}} \int_{\Omega_{d+1}} a(b,y)\mathcal{S}_{h}^{W}( \phi_{j}) (b,y)\overline{\mathcal{S}_{k}^{W}( \varphi_{j}) (b,y)}\,d{\mu}_{\beta}(b,y) \\ \leqslant{}&\frac{1}{\sqrt{\mathcal{C}_{h}\mathcal{C}_{k}}} \int _{\Omega_{d+1}} \big|a(b,y)\big| \\ &\times\Biggl( \sum_{j=1}^{\infty}\big| \mathcal{S}_{h}^{W} ( \phi_{j}) (b,y)\big|^{2} \Biggr)^{\frac{1}{2}} \Biggl( \sum _{j=1}^{\infty} \big|\mathcal{S}_{k}^{W}( \varphi_{j}) (b,y)\big|^{2} \Biggr)^{\frac{1}{2}}\,d{\mu }_{\beta}(b,y) \\ \leqslant{}&\frac{1}{\sqrt{\mathcal{C}_{h}\mathcal {C}_{k}}} \int_{\Omega_{d+1}} \big|a(b,y)\big|\| h_{b,y} \|_{L^{2}_{\beta}(\overline{\mathbb {R}^{d}_{+}})} \| k_{b,y}\|_{L^{2}_{\beta }(\overline{\mathbb {R}^{d}_{+}})} \,d{\mu}_{\beta}(b,y) \\ \leqslant{}&\frac{1}{\sqrt{\mathcal {C}_{h}\mathcal{C}_{k}}}\|a\|_{L^{1}_{\mu_{\beta}}(\Omega_{d+1})}.\end{aligned} $$

Thus

$$\big\| \mathcal {L}_{h,k}(a)\big\| _{S_{1}} \leq\frac{1}{\sqrt{\mathcal{C}_{h}\mathcal {C}_{k}}}\|a\|_{L^{1}_{\mu_{\beta}}(\Omega_{d+1})}. $$

We now prove that $\mathcal {L}_{h,k}(a)$ satisfies the first member of (3.12). Indeed, from (3.13) we have

$$\begin{aligned} \big|\widetilde{a}(b,y)\big|&=\big| \bigl\langle \mathcal {L}_{h,k}(a)( h_{b,y}), k_{b,y} \bigr\rangle _{L^{2}_{\beta}(\overline{\mathbb {R}^{d}_{+}})}\big| \\ &=\Bigg| \sum_{j=1}^{\infty} s_{j}\langle h_{b,y},\phi_{j}\rangle_{L^{2}_{\beta}(\overline{\mathbb {R}^{d}_{+}})}\langle\varphi _{j},k_{b,y}\rangle_{L^{2}_{\beta}(\overline{\mathbb {R}^{d}_{+}})}\Bigg| \\ &\leqslant\frac{1}{2} \sum_{j=1}^{\infty} s_{j} \bigl(\big|\langle h_{b,y},\phi_{j} \rangle_{L^{2}_{\beta}(\overline{\mathbb {R}^{d}_{+}})}\big|^{2}+\big|\langle k_{b,y}, \varphi_{j}\rangle_{L^{2}_{\beta }(\overline{\mathbb {R}^{d}_{+}})}\big|^{2} \bigr).\end{aligned} $$

Then, using Fubini’s theorem, we obtain

$$\begin{aligned} \int_{\Omega_{d+1}}\big|\widetilde{a}(b,y)\big|\,d{\mu}_{\beta}(b,y)={}& \frac {1}{2} \sum_{j=1}^{\infty} s_{j} \biggl( \int_{\Omega_{d+1}}\big|\langle h_{b,y}, \phi_{j} \rangle_{L^{2}_{\beta}(\overline{\mathbb {R}^{d}_{+}})}\big|^{2}\,d{\mu}_{\beta}(b,y) \\ &+ \int_{\Omega_{d+1}} \big|\langle k_{b,y},\varphi_{j} \rangle_{L^{2}_{\beta}(\overline{\mathbb {R}^{d}_{+}})}\big|^{2}\,d{\mu}_{\beta}(b,y) \biggr).\end{aligned} $$

Thus, applying Plancherel’s identity for $\mathcal{S}_{h}^{W}$ and $\mathcal{S}_{k}^{W}$, we get

$$\int_{\Omega_{d+1}}\big|\widetilde{a}(b,y)\big|\,d{\mu}_{\beta}(b,y) \leq \frac{\mathcal{C}_{h}+\mathcal{C}_{k}}{2} \sum_{j=1}^{\infty} s_{j} = \frac{\mathcal{C}_{h}+\mathcal{C}_{k}}{2} \big\| \mathcal {L}_{h,k}(a)\big\| _{S_{1}}. $$

The proof is complete. □

#### Corollary 3.1

*For* *a* *in* $L^{1}_{\mu_{\beta}}(\Omega_{d+1})$, *we have the trace formula*

3.14 $$ \operatorname{tr}\bigl(\mathcal {L}_{h,k}(a)\bigr)=\frac{1}{\sqrt{\mathcal {C}_{h}\mathcal{C}_{k}}} \int_{\Omega_{d+1}} a(b,y) \langle k_{b,y},h_{b,y} \rangle_{L^{2}_{\beta}(\overline{\mathbb {R}^{d}_{+}})}\,d{\mu}_{\beta}(b,y). $$

#### Proof

By Proposition 3.2, $\mathcal {L}_{h,k}(a)\in S_{1}$. Then, using (3.2), we get

$$\begin{aligned} \operatorname{tr}\bigl(\mathcal {L}_{h,k}(a)\bigr)&= \sum_{j=1}^{\infty} \bigl\langle \mathcal {L}_{h,k}(a)(\phi_{j}), \phi_{j} \bigr\rangle _{L^{2}_{\beta}(\overline{\mathbb {R}^{d}_{+}})} \\ &= \sum_{j=1}^{\infty} \frac{1}{\sqrt{\mathcal{C}_{h}\mathcal{C}_{k}}} \int_{\Omega_{d+1}} a(b,y)\langle\phi_{j}, h_{b,y} \rangle_{L^{2}_{\beta}(\overline{\mathbb {R}^{d}_{+}})}\overline{\langle\phi_{j}, k_{b,y} \rangle_{L^{2}_{\beta }(\overline{\mathbb {R}^{d}_{+}})}} \,d{\mu}_{\beta}(b,y) \\ &= \frac{1}{\sqrt{\mathcal{C}_{h}\mathcal{C}_{k}}} \int_{\Omega _{d+1}}\sum_{j=1}^{\infty} \langle\phi_{j}, h_{b,y}\rangle_{L^{2}_{\beta}(\overline{\mathbb {R}^{d}_{+}})}\overline{ \langle\phi_{j}, k_{b,y}\rangle_{L^{2}_{\beta}(\overline{\mathbb {R}^{d}_{+}})}} \,d{ \mu}_{\beta}(b,y) \\ &= \frac{1}{\sqrt{\mathcal{C}_{h}\mathcal{C}_{k}}} \int_{\Omega _{d+1}} \langle k_{b,y}, h_{b,y} \rangle_{L^{2}_{\beta}(\overline{\mathbb {R}^{d}_{+}})} \,d{\mu}_{\beta}(b,y), \end{aligned} $$

and the proof is complete. □

#### Corollary 3.2

*Let* *a* *be in* $L^{p}_{\mu_{\beta}}(\Omega_{d+1})$, $1\leqslant p\leqslant\infty$. *Then*, *the localization operator*

$$\mathcal {L}_{h,k}(a): L^{2}_{\beta} \bigl(\overline{\mathbb {R}^{d}_{+}} \bigr)\longrightarrow L^{2}_{\beta } \bigl(\overline{ \mathbb {R}^{d}_{+}} \bigr) $$

*is in* $S_{p}$, *and*

$$\big\| \mathcal {L}_{h,k}(a)\big\| _{S_{p}}\leqslant \biggl(\frac{1}{\sqrt{\mathcal {C}_{h}\mathcal{C}_{k}}} \biggr)^{\frac{1}{p}}\|a\|_{L^{p}_{\mu_{\beta }}(\Omega_{d+1})}. $$

#### Proof

The result follows from Proposition 3.3 and Theorem 3.2 and by interpolation [17, Theorems 2.10 and 2.11]. □

## Continuous Weinstein wavelet transform and time-frequency concentration

In this section, we suppose that the Weinstein wavelet *h* belongs to $L^{1}_{\beta}(\overline{\mathbb {R}^{d}_{+}}) \cap L^{2}_{\beta}(\overline {\mathbb {R}^{d}_{+}})$.

### Proposition 4.1

*Let* *h* *be a Weinstein wavelet on* $\overline{\mathbb {R}^{d}_{+}}$ *in* $L^{2}_{\beta}(\overline{\mathbb {R}^{d}_{+}})$. *Then*, $\mathcal{S}_{h}^{W}(L^{2}_{\beta}(\overline{\mathbb {R}^{d}_{+}}) ) $ *is a reproducing kernel Hilbert space with kernel*

4.1 $$ \mathcal{K}_{h} \bigl(b',y';b,y \bigr) := \frac{1}{\mathcal{C}_{h}} \int_{\overline{\mathbb {R}^{d}_{+}}}h_{b',y'}(x)\overline{h_{b,y}(x)} \,d\lambda_{\beta}(x). $$

*The kernel is pointwise bounded*:

4.2 $$ \forall \bigl(b',y' \bigr), (b,y ) \in \Omega_{d+1},\quad\big|\mathcal{K}_{h} \bigl(b',x ';b,y \bigr)\big|\leq\frac{\|h\|_{L^{2}_{\beta}(\overline{\mathbb {R}^{d}_{+}})}^{2}}{\mathcal{C}_{h}}. $$

### Proof

Let $f \in L^{2}_{\beta}(\overline{\mathbb {R}^{d}_{+}})$. We have

$$\forall(b,y ) \in\Omega_{d+1},\quad\mathcal{S}_{h}^{W}(f) (b,y) = \int_{\overline{\mathbb {R}^{d}_{+}}}f(x)\overline{h_{b,y}(x)} \,d \lambda_{\beta}(x). $$

Using relation (2.27), we obtain

$$\mathcal{S}_{h}^{W}(f) (b,y) = \frac{1}{\mathcal{C}_{h}} \int_{0}^{\infty} \int_{\overline{\mathbb {R}^{d}_{+}}} \mathcal{S}_{h}^{W}(f) \bigl(b',y' \bigr)\overline{\mathcal{S}_{h}^{W}(h_{b,y}) \bigl(b',y' \bigr)} \,d{\mu}_{\beta} \bigl(b',y' \bigr). $$

Applying Proposition 2.2(iii), we find that, for all $b,b'> 0$ and $y,y' \in\overline{\mathbb {R}^{d}_{+}}$, the function

$$y' \mapsto{\mathcal{S}_{h}^{W}(h_{b,y}) \bigl(b',y' \bigr)} = \frac{1}{\mathcal{C}_{h}} \int_{\overline{\mathbb {R}^{d}_{+}}}h_{b',y'}(x)\overline{h_{b,y}(x)} \,d \lambda_{\beta}(x) $$

belongs to $L^{2}_{\beta}(\overline{\mathbb {R}^{d}_{+}})$. Therefore, the result is obtained. □

In the following we denote:

- $P_{h}: L^{2}_{\mu_{\beta}}(\Omega_{d+1}) \rightarrow L^{2}_{\mu _{\beta}}(\Omega_{d+1})$ is the orthogonal projection from $L^{2}_{\mu_{\beta}}(\Omega_{d+1})$ onto $\mathcal {S}_{h}^{W}(L^{2}_{\beta}(\overline{\mathbb {R}^{d}_{+}}))$.
- $P_{U}: L^{2}_{\mu_{\beta}}(\Omega_{d+1}) \rightarrow L^{2}_{\mu _{\beta}}(\Omega_{d+1})$ is the orthogonal projection on $L^{2}_{\mu _{\beta}}(\Omega_{d+1})$ defined by
  $$\forall v \in L^{2}_{\mu_{\beta}}(\Omega_{d+1}), \quad P_{U}v = \chi_{U}v, $$
  where $\chi_{{{U}}}$ denotes the characteristic function of $U \subset\Omega_{d+1}$ with
  $$0 < \mu_{\beta}(U):= \int_{U}d\mu_{\beta}(b,y) < \infty. $$

We put

$$\|P_{U}P_{h}\|:= \sup \bigl\{ \|P_{U}P_{h}v \|_{L^{2}_{\mu_{\beta }}(\Omega_{d+1})}: v\in L^{2}_{\mu_{\beta}}(\Omega_{d+1}), \|v\|_{L^{2}_{\mu_{\beta}}(\Omega_{d+1})}=1 \bigr\} . $$

We further prove the concentration of $\mathcal{S}_{h}^{W}(f)$ in small sets.

### Proposition 4.2

*Let* *h* *be a Weinstein wavelet*, *and let* $U \subset\Omega_{d+1} $ *with*

$$\mu_{\beta}(U) < \frac{\mathcal{C}_{h}}{\|h\|^{2}_{L^{2}_{\beta }(\overline{\mathbb {R}^{d}_{+}})}}. $$

*Then*, *for all* $f\in L^{2}_{\beta}(\overline{\mathbb {R}^{d}_{+}})$, *we have*

4.3 $$ \big\| \mathcal{S}_{h}^{W}(f)-\chi_{{{U}}} \mathcal{S}_{h}^{W}(f)\big\| _{L^{2}_{\mu_{\beta}}(\Omega_{d+1})} \geq \sqrt{ \mathcal{C}_{h}}\sqrt{1-\frac{\|h\|^{2}_{L^{2}_{\beta }(\overline{\mathbb {R}^{d}_{+}})}}{\mathcal{C}_{h}} \mu_{\beta}(U)}\|f\|_{L^{2}_{\beta}(\overline{\mathbb {R}^{d}_{+}})}, $$

*where* $\chi_{{{U}}}$ *denotes the characteristic function of* *U*.

### Proof

From Plancherel’s formula (Theorem 2.1) we have

4.4 $$ \mathcal{C}_{h}\|f\|^{2}_{L^{2}_{\beta}(\overline {\mathbb {R}^{d}_{+}})} = \big\| \mathcal{S}_{h}^{W}(f)\big\| ^{2}_{L^{2}_{\mu_{\beta }}(\Omega_{d+1})} = \big\| \mathcal{S}_{h}^{W}(f)\big\| ^{2}_{L^{2}_{\mu _{\beta}}(U)} + \big\| \mathcal{S}_{h}^{W}(f)\big\| ^{2}_{L^{2}_{\mu_{\beta }}(U^{c})}. $$

On the other hand, from relation (2.24) we have

4.5 $$ \begin{aligned} \int_{U}\big|\mathcal{S}_{h}^{W}(f) (b,y)\big|^{2}\,d\mu_{\beta}(b,y) &\leq\big\| \mathcal{S}_{h}^{W}(f) \big\| _{L^{\infty}_{\mu_{\beta}}(\Omega _{d+1})}^{2} \mu_{\beta}(U) \\&\leq\mu_{\beta}(U) \|f\|_{L^{2}_{\beta }(\overline{\mathbb {R}^{d}_{+}})}^{2}\|h\|_{L^{2}_{\beta}(\overline{\mathbb {R}^{d}_{+}})}^{2}.\end{aligned} $$

Thus, using (4.4) and (4.5), we deduce the result. □

### Remark 4.1

If $\mathcal{S}_{h}^{W}(f)$ is supported in *U* and $\mu_{\beta}(U) < \frac{\mathcal{C}_{h}}{\|h\|^{2}_{L^{2}_{\beta }(\overline{\mathbb {R}^{d}_{+}})}}$, then $f = 0$.

We further prove the concentration of $\mathcal{S}_{h}^{W}(f)$ in arbitrary sets of finite measures.

### Theorem 4.1

*Let* *h* *be a Weinstein wavelet*, *and let* $U \subset\Omega_{d+1}$ *with* $0 < \mu_{\beta}(U) < \infty $. *If* $P_{h}(L^{2}_{\mu_{\beta}}(\Omega_{d+1})) \cap P_{U}(L^{2}_{\mu_{\beta}}(\Omega_{d+1})) = \{0\}$, *then there exists a constant* $C:= C(h,U) > 0$ *such that*, *for all* $f\in L^{2}_{\beta}(\overline{\mathbb {R}^{d}_{+}})$,

4.6 $$ \big\| \mathcal{S}_{h}^{W}(f)-\chi_{{{U}}} \mathcal{S}_{h}^{W}(f)\big\| _{L^{2}_{\mu_{\beta}}(\Omega_{d+1})} \geq C\|f \|_{L^{2}_{\beta}(\overline{\mathbb {R}^{d}_{+}})}. $$

For the proof of this theorem, we need the following lemma.

### Lemma 4.1

([20])

*Let* $\mathcal{H}_{1}$ *and* $\mathcal{H}_{2}$ *be two closed subspaces of a Hilbert space* $\mathcal{H}$ *such that* $\mathcal{H}_{1} \cap\mathcal{H}_{2} = \{0\}$. *Let* $P_{\mathcal{H}_{1}}$ *and* $P_{\mathcal{H}_{2}}$ *denote the corresponding orthogonal projections*, *and assume that the product* $P_{\mathcal{H}_{1}} P_{\mathcal{H}_{2}}$ *is a compact operator*. *Then*, *there exists a constant* $C > 0$ *such that*, *for* $f \in\mathcal{H}$,

4.7 $$ \|P_{\mathcal{H}_{1}^{\bot}}f\|_{\mathcal{H}}+ \| P_{\mathcal {H}_{2}^{\bot}}f \|_{\mathcal{H}} \geq C \|f\|_{\mathcal{H}}. $$

### Proof of Theorem 4.1

Define $\mathcal{H}_{1}$ and $\mathcal{H}_{2}$ by

$$\mathcal{H}_{1}:= P_{U} \bigl(L^{2}_{\mu_{\beta}}( \Omega_{d+1}) \bigr), \qquad\mathcal{H}_{2}:= P_{h} \bigl(L^{2}_{\mu_{\beta}}(\Omega_{d+1}) \bigr). $$

Proceeding as in [21], we prove that

4.8 $$ \begin{aligned}[b] \|P_{U}P_{h}\|_{HS}&:= \biggl( \int_{\Omega_{d+1}\times\Omega _{d+1}}\big|\chi_{U}(b,y)\big|^{2} \big| \mathcal{K}_{h} \bigl(b',y';b,y \bigr)\big|^{2}\,d\mu_{\beta} \bigl(b',y' \bigr)\,d\mu_{\beta }(b,y) \biggr)^{\frac{1}{2}} \\&\leq\frac{\|h\|_{L^{2}_{\beta }(\overline{\mathbb {R}^{d}_{+}})}}{\sqrt{\mathcal{C}_{h}}} \sqrt{\mu_{\beta}(U)}< \infty.\end{aligned} $$

Hence, $P_{U}P_{h}$ is a Hilbert-Schmidt operator and therefore compact. Now, Lemma 4.1 implies the existence of a constant $C > 0$ such that (4.7) holds for $P_{\mathcal{H}_{1}}:= P_{U}$ and $P_{\mathcal{H}_{2}}:= P_{h}$. Since

$$P_{\mathcal{H}_{2}^{\bot}} \bigl(\mathcal{S}_{h}^{W}(f) \bigr) = (\mathit{Id} - P_{h})\mathcal{S}_{h}^{W}(f) = 0, $$

this leads to (4.6). □

### Definition 4.1

Let *h* be a Weinstein wavelet, and let $U \subset\Omega_{d+1} $ such that $0 < \mu_{\beta}(U) < \infty$. Then

1. We say that *U* is weakly annihilating if any function $f \in L^{2}_{\beta}(\overline{\mathbb {R}^{d}_{+}})$ vanishes when its Weinstein wavelet transform $\mathcal{S}_{h}^{W}(f)$ with respect to the Weinstein wavelet *h* is supported in *U*.
2. We say that *U* is strongly annihilating if there exists a constant $C_{ \beta} (U) > 0$ such that, for every function $f \in L^{2}_{\beta}(\overline{\mathbb {R}^{d}_{+}})$,
  4.9 $$ C_{ \beta} (U)\big\| \mathcal{S}_{h}^{W}(f)- \chi_{{{U}}}\mathcal{S}_{h}^{W}(f)\big\| _{L^{2}_{\mu_{\beta}}(\Omega_{d+1})} \geq\|f\|_{L^{2}_{\beta}(\overline{\mathbb {R}^{d}_{+}})}\|h\|_{L^{2}_{\beta }(\overline{\mathbb {R}^{d}_{+}})}. $$
  The constant $C_{ \beta} (U)$ is called the annihilation constant of *U*.

The analogue of this definition was introduced by Ghobber and Omri [22] in the cadre of windowed Hankel transform.

### Remark 4.2

1. It is clear that every strongly annihilating set is also a weakly.
2. From Proposition 4.2 we see that any set $U \subset \Omega_{d+1} $ with $\mu_{\beta}(U) < \frac{\mathcal {C}_{h}}{\|h\|^{2}_{L^{2}_{\beta}(\overline{\mathbb {R}^{d}_{+}})}}$ is strongly annihilating.
3. As the operator $P_{U}P_{h}$ is Hilbert-Schmidt and hence compact, from [23] we have that if *U* is weakly annihilating, then it is also strongly annihilating.
4. If $\|P_{U}P_{h}\| < 1$, then, for all $f \in L^{2}_{\beta }(\overline{\mathbb {R}^{d}_{+}})$,
  4.10 $$ \frac{1}{\sqrt{1-\|P_{U}P_{h}\|^{2}}}\big\| \chi_{{{U^{c}}}}\mathcal {S}_{h}^{W}(f) \big\| _{L^{2}_{\mu_{\beta}}(\Omega_{d+1})} \geq\sqrt{\mathcal {C}_{h}}\|f\|_{L^{2}_{\beta}(\overline{\mathbb {R}^{d}_{+}})}\|h \|_{L^{2}_{\beta}(\overline{\mathbb {R}^{d}_{+}})}. $$
5. Following [23, p.88], we have that if *U* is strongly annihilating, then $\|P_{U}P_{h}\| < 1$.

In the following, we prove the Benedicks uncertainty principle for the Weinstein wavelet transform under some condition on the Weinstein wavelet. We note that the Benedicks-type uncertainty principle for the windowed Hankel transform was studied by Ghobber and Omri [22].

### Theorem 4.2

*Let* *h* *be a Weinstein wavelet such that*

4.11 $$ \int_{\{\xi\in\overline{\mathbb {R}^{d}_{+}}:\mathcal{F}_{W}(\bar{h})(\xi) \neq0\}}\,d\lambda_{\beta }(\xi) < \infty. $$

*For any subset* $U \subset\Omega_{d+1}$ *such that*

$$\int_{\overline{\mathbb {R}^{d}_{+}}}\chi_{U}(b,y)\,d\lambda_{\beta}(y) < \infty $$

*for almost every* $b > 0$, *we have*

4.12 $$ P_{h} \bigl(L^{2}_{\mu_{\beta}}(\Omega_{d+1}) \bigr) \cap P_{U} \bigl(L^{2}_{\mu _{\beta}}( \Omega_{d+1}) \bigr) = \{0\}. $$

### Proof

Let $F \in P_{h}(L^{2}_{\mu_{\beta}}(\Omega_{d+1})) \cap P_{U}(L^{2}_{\mu_{\beta}}(\Omega_{d+1})) \setminus\{0\}$. Then by the definition of the operators $P_{U}$ and $P_{h}$ there exists a function $f \in L^{2}_{\beta}(\overline{\mathbb {R}^{d}_{+}})$ such that $F = \mathcal{S}_{h}^{W}(f)$ and $\operatorname{supp} F \subset U$.

Let $b > 0$ be such that $\displaystyle\int_{\overline{\mathbb {R}^{d}_{+}}}\chi_{{{U}}}(b,y)\,d\lambda_{\beta}(y) < \infty$. Consider the function $F_{b}$ defined by

$$F_{b}(y) = \mathcal{S}_{h}^{W}(f) (b,y), \quad y \in\overline{\mathbb {R}^{d}_{+}}. $$

Then we have

$$\operatorname{supp} F_{b} \subset \bigl\{ y\in\overline{ \mathbb {R}^{d}_{+}}: (b,y) \in U \bigr\} $$

and

$$\int_{\operatorname{supp} F_{b}}\,d\lambda_{\beta}(\xi) < \infty. $$

On the other hand, using (2.23) and hypothesis (4.11), we get

$$\int_{ \{\xi\in\overline{\mathbb {R}^{d}_{+}}:\mathcal{F}_{W}(F_{b})(\xi ) \neq0\}}d\lambda_{\beta}(\xi) < \infty. $$

Using Proposition 2.3, we obtain that $F_{b} = 0$ for every $b > 0$, and hence $F = 0$. □

Consequently, we obtain the following improvement.

### Corollary 4.1

*Let* *h* *be a Weinstein wavelet on* $\overline{\mathbb {R}^{d}_{+}}$ *in* $L^{2}_{\beta}(\overline{\mathbb {R}^{d}_{+}})$ *such that*

$$\int_{\{\xi\in\overline{\mathbb {R}^{d}_{+}}:\mathcal{F}_{W}(\bar {h})(\xi) \neq0\}}d\lambda_{\beta}(\xi) < \infty. $$

*Then*, *for any subset* $U \subset\Omega_{d+1} $ *such that* $0 < \mu _{\beta}(U) < \infty$, *there exists a constant* $C:= C(h,U) > 0$ *such that*, *for all* $f\in L^{2}_{\beta}(\overline{\mathbb {R}^{d}_{+}})$, *we have*

4.13 $$ \big\| \mathcal{S}_{h}^{W}(f)-\chi_{{{U}}} \mathcal{S}_{h}^{W}(f)\big\| _{L^{2}_{\mu_{\beta}}(\Omega_{d+1})} \geq C\|f \|_{L^{2}_{\beta}(\overline{\mathbb {R}^{d}_{+}})}. $$

Now we will derive a sufficient condition by means of which we can recover a signal *F* belonging to $L^{ 2}_{\mu_{\beta}}(\Omega_{d+1})$ from the knowledge of its truncated version following the Donoho-Stark criterion [24].

Let *h* be a Weinstein wavelet function. A signal $F \in L^{ 2}_{\mu _{\beta}}(\Omega_{d+1})$ is transmitted to a receiver who knows that $F \in\mathcal{S}_{h}^{W}(L^{2}_{\beta}(\overline{\mathbb {R}^{d}_{+}}))$. Suppose that the observation of F is corrupted by a noise $n \in L^{ 2}_{\mu_{\beta}}(\Omega _{d+1})$ (which is nonetheless assumed to be small) and unregistered values on $U \in\Omega_{d+1}$. Thus, the observable function *r* satisfies

4.14 $$ r(b,y) = \left \{ \textstyle\begin{array}{l@{\quad}l} F(b,y) + n(b,y) & {\text{if }} (b,y) \in U^{c}, \\ 0& {\text{if }} (b,y) \in U. \end{array}\displaystyle \right . $$

Here we have assumed without loss of generality that $n = 0$ on *U*. Equivalently,

4.15 $$ r = (\mathit{Id} - P_{U})F + n. $$

We say that *F* can be stably reconstructed from *r* if there exist a linear operator

$$L_{U,h}:L^{ 2}_{\mu_{\beta}}(\Omega_{d+1}) \rightarrow L^{ 2}_{\mu _{\beta}}(\Omega_{d+1}) $$

and a constant $C(U,h)$ such that

4.16 $$ \big\| F - L_{U,h}(r)\big\| _{L^{ 2}_{\mu_{\beta}}(\Omega_{d+1})} \leq C(U,h)\|n \|_{L^{ 2}_{\mu_{\beta}}(\Omega_{d+1})}. $$

Proceeding as in [24], it is easy to prove the following:

### Proposition 4.3

*Let* *h* *be a Weinstein wavelet function such that*

$$\int_{\{\xi\in\overline{\mathbb {R}^{d}_{+}}:\mathcal{F}_{W}(\bar {h})(\xi) \neq0\}}d\lambda_{\beta}(\xi) < \infty. $$

*Let* $U\subset\Omega_{d+1}$ *with* $0 < \mu_{\beta}(U) < \infty$. *Then* *F* *can be stably reconstructed from* *r*. *The constant* $C(U,h)$ *in* (4.16) *is not larger than* $(1-\|P_{U}P_{h}\|)^{-1}$.

The identity

$$Q = (\mathit{Id} - P_{U}P_{h})^{-1} = \sum _{j=0}^{\infty}(P_{U}P_{h})^{j} $$

suggests an algorithm for computing $Q(r)$. Finally, using a method similar to that in [24], we give an algorithm for computing $L_{U,h}$. Indeed, put

$$F_{k} = \sum_{j=0}^{k}(P_{U}P_{h})^{j}r. $$

Then $F_{k} \rightarrow Q(r)$ as $k \to\infty$. Now

4.17 $$ \begin{gathered} F_{0} = r, \\ F_{1} = r + P_{U}P_{h}F_{0}, \\ F_{2} = r + P_{U}P_{h}F_{1}, \\ \ldots \end{gathered} $$

and so on. The iteration converges at a geometric rate to the fixed point

$$F = r + P_{U}P_{h}F. $$

Algorithms of type (4.17), have been applied to a host of problems in signal recovery; see [24] and others.

## Heisenberg-type uncertainty inequalities for the Weinstein wavelet transform

In this section, we establish many Heisenberg-type uncertainty inequalities for the Weinstein wavelet transform.

### Proposition 5.1

*We assume that the Weinstein wavelet* *h* *satisfies* ${\| h\|} _{{L^{2}_{\beta}(\overline{\mathbb {R}^{d}_{+}})}}= 1 $.

*Let* $s > 0$. *Then we have the following uncertainty inequalities*.

1. *There exists a constant* $C_{1}(\beta,s) > 0$ *such that*, *for all* $f \in L^{2}_{\beta}(\overline{\mathbb {R}^{d}_{+}})$,
  5.1 $$ \bigg\| \bigg\| \biggl(\frac{1}{b},y \biggr)\bigg\| ^{s} \mathcal{S}_{h}^{W}(f)\bigg\| _{L^{2}_{\mu_{\beta}}(\Omega_{d+1})} \geq C_{1}(\beta,s){\| f\|} _{{L^{2}_{\beta}(\overline{\mathbb {R}^{d}_{+}})}}. $$
2. *There exists a constant* $C_{2}(\beta,s) > 0$ *such that*, *for all* $f \in L^{2}_{\beta}(\overline{\mathbb {R}^{d}_{+}})$,
  5.2 $$ \big\| \|y\|^{s}\mathcal{S}_{h}^{W}(f)\big\| _{L^{2}_{\mu _{\beta}}(\Omega_{d+1})} \big\| |b|^{-s}\mathcal{S}_{h}^{W}(f)\big\| _{L^{2}_{\mu_{\beta}}(\Omega_{d+1})} \geq C_{2}(\beta,s){\| f\|} _{{L^{2}_{\beta}(\overline{\mathbb {R}^{d}_{+}})}}^{2}. $$
3. *There exists a constant* $C_{3}(\beta,s) > 0$ *such that*, *for all* $f \in L^{2}_{\beta}(\overline{\mathbb {R}^{d}_{+}})$ *and every measurable subset* *U* *of finite measure* $0 < \mu_{\beta}(U) < \infty$,
  5.3 $$ \big\| \mathcal{S}_{h}^{W}(f)\big\| _{L^{2}_{\mu_{\beta}}(\Omega_{d+1})} \leq C_{3}(\beta,s)\sqrt{\mu_{\beta}(U)}\bigg\| \bigg\| \biggl( \frac {1}{b},y \biggr)\bigg\| ^{s}\mathcal{S}_{h}^{W}(f)\bigg\| _{L^{2}_{\mu_{\beta }}(\Omega_{d+1})}. $$

### Proof

(1) Let $r \in(0,1]$ be such that $\mu_{\beta}(U_{r}) < \mathcal {C}_{h}$, where

$$U_{r} = \biggl\{ (b,y) \in\Omega_{d+1}: \bigg\| \biggl( \frac{1}{b},y \biggr)\bigg\| < r \biggr\} . $$

From Proposition 4.2 we have

$$\begin{aligned} \|f\|_{L^{2}_{\beta}(\overline{\mathbb {R}^{d}_{+}})}^{2} &\leq \frac {1}{{\mathcal{C}_{h}-\mu_{\beta}(U_{r})}} \int_{U_{r}^{c}}\big|\mathcal {S}_{h}^{W}(f) (b,y)\big|^{2}\,d\mu_{\beta} (b,y) \\ &\leq\frac{1}{r^{2s}(\mathcal{C}_{h}-\mu_{\beta }(U_{r}))} \int_{\|(\frac{1}{b},y)\|\geq r }\bigg\| \biggl(\frac {1}{b},y \biggr) \bigg\| ^{2s}\big|\mathcal{S}_{h}^{W}(f) (b,y)\big|^{2} \,d\mu_{\beta} (b,y) \\ &\leq\frac{1}{r^{2s}(\mathcal{C}_{h}-\mu_{\beta }(U_{r}))}\bigg\| \bigg\| \biggl(\frac{1}{b},y \biggr) \bigg\| ^{s}\mathcal{S}_{h}^{W}(f) \bigg\| _{L^{2}_{\mu_{\beta}}(\Omega_{d+1})}^{2}. \end{aligned} $$

Thus we have obtained the result with $C_{1}(\beta,s):= r^{s}\sqrt {\mathcal{C}_{h}-\mu_{\beta}(U_{r})}$.

Now we will prove (2). Indeed, using the fact that $\|(\frac {1}{b},y)\|^{s} \leq2^{s}(|b|^{-s}+\|y\|^{s})$ in (5.1), we get

$$\big\| \|y\|^{s}\mathcal{S}_{h}^{W}(f)\big\| _{L^{2}_{\mu _{\beta}}(\Omega_{d+1})}^{2}+ \big\| |b|^{-s}\mathcal {S}_{h}^{W}(f)\big\| _{L^{2}_{\mu_{\beta}}(\Omega_{d+1})}^{2} \geq \frac{C_{1}^{2}(\beta,s)}{2^{2s}}{\| f\|} _{{L^{2}_{\beta }(\overline{\mathbb {R}^{d}_{+}})}}^{2}. $$

Replacing *f* by $\delta_{t}f:= \frac{1}{t^{\beta+\frac {d+1}{2}}}f(\frac{\cdot}{t})$ in the previous inequality, by (2.25) and by a suitable change of variables we obtain:

$$t^{2s}\big\| \|y\|^{s}\mathcal{S}_{h}^{W}(f)\big\| _{L^{2}_{\mu_{\beta}}(\Omega_{d+1})}^{2}+ t^{-2s}\big\| |b|^{-s} \mathcal{S}_{h}^{W}(f)\big\| _{L^{2}_{\mu_{\beta }}(\Omega_{d+1})}^{2} \geq\frac{C_{1}^{2}(\beta,s)}{2^{2s}}{\| f\|} _{{L^{2}_{\beta }(\overline{\mathbb {R}^{d}_{+}})}}^{2}. $$

Then (5.2) follows by minimizing the left-hand side of that inequality over $t>0$.

Now we will prove (3). Indeed, using the estimates

$$\big\| \mathcal{S}_{h}^{W}(f)\big\| _{L^{2}_{\mu_{\beta}}(U_{r})} \leq\sqrt{ \mu_{\beta}(U)} \big\| \mathcal{S}_{h}^{W}(f) \big\| _{L^{\infty}_{\mu _{\beta}}(\Omega_{d+1})} $$

and

$$\big\| \mathcal{S}_{h}^{W}(f)\big\| _{L^{\infty}_{\mu_{\beta}}(\Omega _{d+1})} \leq\|f \|_{L^{2}_{\beta}(\overline{\mathbb {R}^{d}_{+}})}, $$

we get

$$\big\| \mathcal{S}_{h}^{W}(f)\big\| _{L^{2}_{\mu_{\beta}}(U_{r})} \leq\sqrt{ \mu_{\beta}(U)} \|f\|_{L^{2}_{\beta}(\overline{\mathbb {R}^{d}_{+}})}. $$

Finally, the result immediately follows from (5.1). □

Now we recall the uncertainty principle of Heisenberg type for the Weinstein transform.

### Proposition 5.2

([25])

*For* $s,t > 0$, *there exists a positive constant* $C(\beta,s,t)$ *such that*, *for every* $f \in L^{2}_{\beta}(\overline{\mathbb {R}^{d}_{+}})$, *we have the inequality*

5.4 $$ \big\| \|\xi\|^{s}{\mathcal {F}}_{W}(f) \big\| _{L^{2}_{\beta}(\overline{\mathbb {R}^{d}_{+}})}^{\frac{t}{s+t}} \big\| \| y\|^{t}f\big\| _{L^{2}_{\beta}(\overline{\mathbb {R}^{d}_{+}})}^{\frac{s}{s+t}} \geq C(\beta,s,t) \|f\|_{L^{2}_{\beta }(\overline{\mathbb {R}^{d}_{+}})}. $$

### Theorem 5.1

*Let* $s,t > 0$, *and let* *h* *be a Weinstein wavelet on* $\overline{\mathbb {R}^{d}_{+}}$ *in* $L^{2}_{\beta}(\overline{\mathbb {R}^{d}_{+}})$. *Then*, *for all* *f* *in* $L^{2}_{\beta}(\overline{\mathbb {R}^{d}_{+}})$, *we have the inequality*

5.5 $$ \big\| \|y\|^{s}\mathcal{S}_{h}^{W}(f)\big\| _{L^{2}_{\mu _{\beta}}(\Omega_{d+1})}^{\frac{t}{t+s}} \big\| \|\xi\|^{t}{\mathcal {F}}_{W}(f) \big\| _{L^{2}_{\beta}(\overline{\mathbb {R}^{d}_{+}})}^{\frac {s}{s+t}} \geq C(\beta,s,t) \mathcal{C}_{h}^{\frac{t}{2(s+t)}} \|f\| _{L^{2}_{\beta}(\overline {\mathbb {R}^{d}_{+}})}. $$

### Proof

Let us assume the nontrivial case that both integrals on the left-hand side of (5.5) are finite. We get from the admissibility condition (2.15) for *h* that

$$\int_{0}^{\infty} \int_{\overline{\mathbb {R}^{d}_{+}}}\|\xi\|^{2t} \big| \mathcal{F}_{W}(h) (b\xi)\big|^{2}\big|\mathcal{F}_{W}(f) ( \xi)\big|^{2} \frac{d\lambda_{\beta}(\xi) \,da}{a} = \mathcal{C}_{h} \int_{\overline{\mathbb {R}^{d}_{+}}}\|\xi\|^{2t}\big|\mathcal{F}_{W}(f) ( \xi)\big|^{2} \,d\lambda_{\beta}(\xi). $$

Using relation (2.23), we obtain

5.6 $$ \int_{0}^{\infty} \int_{\overline{\mathbb {R}^{d}_{+}}}\|\xi \|^{2t}\big|\mathcal{F}_{W} \bigl(\mathcal{S}_{h}^{W}(f) (b,\cdot) \bigr) ( \xi)\big|^{2} \,d{\mu}_{\beta}(b,\xi) = \mathcal{C}_{h} \int_{\overline{\mathbb {R}^{d}_{+}}}\|\xi\|^{2t}\big|\mathcal {F}_{W}(f) ( \xi)\big|^{2} \,d\lambda_{\beta}(\xi). $$

Moreover, using (5.4), we get

$$\begin{gathered} \forall b > 0,\quad \biggl( \int_{\overline{\mathbb {R}^{d}_{+}}}\|\xi\|^{2t}\big| { \mathcal{F}}_{W} \bigl(\mathcal{S}_{h}^{W}(f) (b,\cdot) \bigr) ( \xi)\big|^{2}\,d\lambda_{\beta}(\xi) \biggr)^{\frac{s}{s+t}}\\ \phantom{\forall b > 0,\quad}\qquad\times{} \biggl( \int_{\overline{\mathbb {R}^{d}_{+}}}\|y\|^{2s}\big|\mathcal{S}_{h}^{W}(f) (b,y)\big|^{2}\,d\lambda_{\beta}(y) \biggr)^{\frac{t}{s+t}} \\\phantom{\forall b > 0,\quad}\quad\geq C^{2}(\beta,s,t) \int_{\overline{\mathbb {R}^{d}_{+}}}\big|\mathcal {S}_{h}^{W}(f) (b,y )\big|^{2}\,d\lambda_{\beta}(y). \end{gathered}$$

Integrating with respect to $\frac{db}{b^{2\beta+d+2}}$, we obtain

$$\begin{gathered} \int_{0}^{\infty} \biggl[ \biggl( \int_{\overline{\mathbb {R}^{d}_{+}}}\| \xi \|^{2t}\big| {\mathcal{F}}_{W} \bigl(\mathcal{S}_{h}^{W}(f) (b,\cdot) \bigr) ( \xi)\big|^{2}\,d\lambda_{\beta}(\xi) \biggr)^{\frac{s}{s+t}} \\ \qquad{}\times\biggl( \int_{\overline{\mathbb {R}^{d}_{+}}}\|y\| ^{2s}\big|\mathcal {S}_{h}^{W}(f) (b,y)\big|^{2}\,d\lambda_{\beta}(y) \biggr)^{\frac{t}{s+t}} \biggr] \frac{db}{b^{2\beta+d+2}} \\\quad\geq C^{2}(\beta,s,t) \int_{0}^{\infty} \int_{\overline{\mathbb {R}^{d}_{+}}}\big| \mathcal{S}_{h}^{W}(f) (b,y )\big|^{2}\,d \lambda_{\beta}(y) \frac{db}{b^{2\beta+d+2}}. \end{gathered}$$

The left-hand side of this inequality may be estimated from above using Hölder’s inequality. The right-hand side can be rewritten by Plancherel’s formula for $\mathcal{S}_{h}^{W}$. Therefore, from (5.6) we get

$$\begin{aligned}& \biggl( \int_{0}^{\infty} \int_{\overline{\mathbb {R}^{d}_{+}}}\|\xi \|^{2t}\big| {\mathcal{F}}_{W} \bigl(\mathcal{S}_{h}^{W}(f) (b,\cdot) \bigr) ( \xi)\big|^{2}\,d{\mu}_{\beta}(b,\xi) \biggr)^{\frac{s}{s+t}} \\& \qquad{}\times\biggl( \int_{0}^{\infty} \int_{\overline{\mathbb {R}^{d}_{+}}}\|y \|^{2s}\big|\mathcal{S}_{h}^{W} (f) (b,y)\big|^{2}\,d{\mu}_{\beta}(b,y) \biggr)^{\frac{t}{s+t}} \\& \quad= \mathcal{C}_{h}^{\frac{s}{s+t}} \biggl( \int_{\overline{\mathbb {R}^{d}_{+}}}\|\xi \|^{2t}\big| {\mathcal{F}}_{W} \bigl(f(\xi) \bigr)\big|^{2} \,d\lambda_{\beta}(\xi) \biggr)^{\frac{s}{s+t}}\\& \qquad{}\times \biggl( \int_{0}^{\infty} \int_{\overline{\mathbb {R}^{d}_{+}}}\|y \|^{2s}\big|\mathcal{S}_{h}^{W}(f) (b,y)\big|^{2}\,d{\mu}_{\beta}(b,y) \biggr)^{\frac{t}{s+t}} \\& \quad\geq C^{2}(\beta,s,t) \mathcal{C}_{h}\|f \|_{L^{2}_{\beta}(\overline {\mathbb {R}^{d}_{+}})}^{2}. \end{aligned}$$

This proves the result. □

## Heisenberg-type uncertainty inequalities for the modified Weinstein wavelet transform

There is no uncertainty principle of Heisenberg type for $\mathcal {S}_{h}^{W} (f)(b,y)$ with respect to *b*.

For this, we consider the Weinstein wavelet *h* defined by relation (2.16) and introduce the modified Weinstein continuous wavelet transform $\widetilde{\mathcal{S}_{h}^{W} }$ given by

6.1 $$ \widetilde{\mathcal{S}_{h}^{W} }f(b,y) = b^{2\beta+d+1} \mathcal{F}_{W}^{-1} \bigl( \mathcal{F}_{W}f(b\xi)\overline{\mathcal{F}_{W}(h) (\xi)} \bigr) (y), \quad(b,y) \in\Omega_{d+1}. $$

Using this transform, we obtain the following theorem.

### Theorem 6.1

*For* $s,t > 0$ *and every* *f* *in* $L^{2}_{\beta}(\overline{\mathbb {R}^{d}_{+}})$, *we have*

6.2 $$ \big\| b^{t}\widetilde{\mathcal{S}_{h}^{W}}f\big\| _{L^{2}_{\mu_{\beta}}(\Omega_{d+1})} ^{\frac{s}{s+t}}\big\| \|y\|^{s}f\big\| _{L^{2}_{\beta }(\overline{\mathbb {R}^{d}_{+}})} ^{\frac{t}{s+t}} \geq C(\beta,s,t) \bigl( {\mathcal{M} \bigl(e^{-y^{2}} \bigr) (2t-4)} \bigr)^{\frac{s}{2(s+t)}} \|f\| _{L^{2}_{\beta}(\overline{\mathbb {R}^{d}_{+}})}, $$

*where*

$$\mathcal{M}: H \mapsto\mathcal{M}(H) (y) = \int_{0}^{\infty}H(r) \frac{dr}{r^{y+1}} $$

*is the classical Mellin transform*.

### Proof

In the following, we assume that

$$\int_{0}^{\infty} \int_{\overline{\mathbb {R}^{d}_{+}}}b^{2t}\big| \widetilde{\mathcal{S}_{h}^{W}}f(b,y)\big|^{2} \,d \mu_{\beta}(b,y) < \infty\quad{\text{and}}\quad \int_{\overline{\mathbb {R}^{d}_{+}}}\|y \|^{2s}\big|f(y)\big|^{2} \,d \lambda_{\beta}(y) < \infty. $$

Otherwise, (6.2) is trivially satisfied. Using Fubini’s theorem and (2.26), we have

$$\begin{gathered} \int_{\Omega_{d+1}}b^{2t}\big| \bigl(\widetilde{ \mathcal{S}_{h}^{W}}f \bigr) (b,y)\big|^{2} \,d \mu_{\beta}(b,y)\\ \quad= \int_{0}^{\infty}b^{2t} \int_{\overline{\mathbb {R}^{d}_{+}}}b^{2\beta +d+1}\big| \mathcal{F}_{W}f(b \xi)\big|^{2}\big|{\mathcal{F}_{W}(h) ( \xi)}\big|^{2} \,d \lambda_{\beta}(\xi)\frac{db}{b} \\ \quad= \int_{\overline{\mathbb {R}^{d}_{+}}} \int_{0}^{\infty}b^{2t}\big|\mathcal {F}_{W}f(\xi)\big|^{2}\bigg|{\mathcal{F}_{W}(h) \biggl( \frac{\lambda }{b} \biggr)}\bigg|^{2}\frac{db}{b} \,d \lambda_{\beta}(\xi) \\ \quad= \int_{\overline{\mathbb {R}^{d}_{+}}}\Lambda(\xi)\big|\mathcal{F}_{W}(f) (\xi )\big|^{2}{\,d\lambda_{\beta}(\xi)}\end{gathered} $$

with

$$\Lambda(\xi) = \int_{0}^{\infty}b^{2t}\bigg| \mathcal{F}_{W}(h) \biggl(\frac{\lambda }{b} \biggr)\bigg|^{2} \frac{db}{b}. $$

Using (2.16), by simple calculations we get

$$\begin{aligned} \Lambda(\xi) & = \int_{0}^{\infty}b^{2t}\bigg| \mathcal{F}_{W}(h) \biggl(\frac{\lambda }{b} \biggr)\bigg|^{2} \frac{db}{b} \\ & = \biggl( \int_{0}^{\infty}e^{-y^{2}} \frac{dy}{y^{2t-3}} \biggr)\|\xi\|^{2t} \\ &= \bigl( {\mathcal{M} \bigl(e^{-y^{2}} \bigr) (2t-4)} \bigr)\|\xi \|^{2t}. \end{aligned} $$

Thus

$$\begin{gathered} \biggl( \int_{0}^{\infty} \int_{\overline{\mathbb {R}^{d}_{+}}}b^{2t}\big|\widetilde{\mathcal{S}_{h}^{W}}f(b,y)\big|^{2} {\,d\mu_{\beta}(b,y)} \biggr)^{\frac{s}{s+t}} \biggl( \int _{\overline{\mathbb {R}^{d}_{+}}}\|y \|^{2s}\big|f(y)\big|^{2} \,d \lambda_{\beta}(y) \biggr)^{\frac{t}{s+t}} \\ \quad= \bigl( {\mathcal{M} \bigl(e^{-y^{2}} \bigr) (2t-4)} \bigr) ^{\frac{s}{(s+t)}} \\ \quad\quad{}\times \biggl( \int_{\overline{\mathbb {R}^{d}_{+}}}\|\xi\|^{2t}\big| \mathcal{F}_{W}(f) ( \xi)\big|^{2} \,d\lambda_{\beta}(\xi) \biggr)^{\frac{s}{s+t}} \biggl( \int_{\overline{\mathbb {R}^{d}_{+}}}\|y\| ^{2s}\big|f(y)\big|^{2} \,d \lambda_{\beta}(y) \biggr)^{\frac{t}{s+t}}. \end{gathered} $$

Now, the result is obtained from Proposition 5.2. □

### Remark 6.1

(i) It is easy to see that $\mathcal{M} (e^{-y^{2}})(2t-4) < \infty$ if and only if $t < 2$.
(ii) In the classical setting, the notion of the modified continuous wavelet transform was first introduced by Wilczok [20].

### Corollary 6.1

*For* $s,t > 0$ *and* *f* *in* $L^{2}_{\beta}(\overline{\mathbb {R}^{d}_{+}})$, *we have*

$$\begin{gathered} \big\| \|y\|^{s}{\mathcal{S}_{h}^{W}}f\big\| _{L^{2}_{\mu _{\beta}}(\Omega_{d+1})} ^{\frac{t}{s+t}}\big\| b^{t}\widetilde {\mathcal {S}_{h}^{W}}f\big\| _{L^{2}_{\mu_{\beta}}(\Omega_{d+1})} ^{\frac {s}{s+t}}\\\quad \geq C( \beta,s,t)\mathcal{C}_{h}^{\frac{t}{2(s+t)}} \bigl(\mathcal{M} \bigl(e^{-y^{2}} \bigr) (2t-4) \bigr)^{\frac{s}{2(s+t)}} \|f\| _{L^{2}_{\beta}(\overline{\mathbb {R}^{d}_{+}})}.\end{gathered} $$

### Proof

By the preceding we have

$$\begin{gathered} \biggl( \int_{0}^{\infty} \int_{\overline{\mathbb {R}^{d}_{+}}}b^{2t}\big|\widetilde{\mathcal{S}_{h}^{W}}f(b,y)\big|^{2} {\,d\mu_{\beta}(b,y)} \biggr)^{\frac{s}{s+t}} \biggl( \int _{0}^{\infty} \int_{\overline{\mathbb {R}^{d}_{+}}}\|y\|^{2s}\big|{\mathcal {S}_{h}^{W}}f(b,y)\big|^{2} {\,d\mu_{\beta}(b,y)} \biggr)^{\frac{t}{s+t}} \\ \quad= \bigl( {\mathcal{M} \bigl(e^{-y^{2}} \bigr) (2t-4)} \bigr)^{\frac {s}{(s+t)}} \biggl( \int_{\overline {\mathbb {R}^{d}_{+}}}\|\xi\|^{2t}\big|\mathcal{F}_{W}(f) ( \xi)\big|^{2} \,d\lambda_{\beta}(\xi) \biggr)^{\frac{s}{s+t}} \\ \quad\quad{}\times \biggl( \int_{0}^{\infty} \int_{\overline{\mathbb {R}^{d}_{+}}}\|y \|^{2s}\big|{\mathcal{S}_{h}^{W}}f(b,y)\big|^{2} {\,d\mu_{\beta}(b,y)} \biggr)^{\frac{t}{s+t}}. \end{gathered} $$

The result follows from relation (5.5). □

### Corollary 6.2

*For* $s,t > 0$ *and for any* *f* *in* $L^{2}_{\beta}(\overline{\mathbb {R}^{d}_{+}})$, *we have*

$$\begin{gathered} \big\| \big\| (b,y)\big\| ^{s}{\mathcal{S}_{h}^{W}}f\big\| _{L^{2}_{\mu_{\beta}}(\Omega_{d+1})} ^{\frac{t}{s+t}}\big\| \big\| (b,y)\big\| ^{t}\widetilde{ \mathcal{S}_{h}^{W}}f\big\| _{L^{2}_{\mu_{\beta}}(\Omega_{d+1})} ^{\frac{s}{s+t}}\\ \quad\geq C(\beta,s,t)\mathcal{C}_{h}^{\frac{s}{2(s+t)}} \bigl( {\mathcal{M} \bigl(e^{-y^{2}} \bigr) (2t-4)} \bigr)^{\frac{s}{2(s+t)}} \|f\| _{L^{2}_{\beta}(\overline{\mathbb {R}^{d}_{+}})}.\end{gathered} $$

### Proof

The result follows from Corollary 6.1 and the fact that

$$\big\| (b,y)\big\| ^{2s} \geq\|y\|^{2s} \quad{\text{and}} \quad\big\| (b,y) \big\| ^{2t} \geq b^{2t}. $$

 □

In the following, we give the local-type uncertainty principle.

### Corollary 6.3

*For* $s > 0$, *subsets* $U \in\Omega_{d+1}$ *such that* $0 < \mu_{\beta}(U) < \infty $, *and* *f* *in* $L^{2}_{\beta}(\overline{\mathbb {R}^{d}_{+}})$, *we have*

$$\begin{gathered} \int_{\Omega_{d+1}}\chi_{U}(b,y)\big|{\mathcal{S}_{h}^{W}}f(b,y)\big|^{2} \,d\mu_{\beta}(b,y)\\ \quad\leq C_{\beta}(s)\big\| \big\| (b,y) \big\| ^{s}{\mathcal{S}_{h}^{W}}f\big\| _{L^{2}_{\mu_{\beta }}(\Omega_{d+1})} ^{\frac{1}{2}}\big\| \big\| (b,y)\big\| ^{s}\widetilde{\mathcal {S}_{h}^{W}}f\big\| _{L^{2}_{\mu_{\beta}}(\Omega_{d+1})} ^{\frac{1}{2}}, \end{gathered}$$

*where*

$$C_{\beta}(s):=\frac{{\mu_{\beta}(U)}\|h\|^{2}_{L^{2}_{\beta }(\overline{\mathbb {R}^{d}_{+}})}}{C(\beta,s,s)\sqrt{\mathcal{C}_{h} ( {\mathcal{M}(e^{-y^{2}})(2t-4)} )}}. $$

### Proof

From relation (4.5), we have

$$\int_{0}^{\infty} \int_{\overline{\mathbb {R}^{d}_{+}}}\chi_{U}(b,y)\big|{ \mathcal{S}_{h}^{W}}f(b,y)\big|^{2} {\,d \mu_{\beta}(b,y)}\leq{\mu_{\beta}(U)}\|h\|^{2}_{L^{2}_{\beta }(\overline{\mathbb {R}^{d}_{+}})} \|f\|^{2}_{L^{2}_{\beta}(\overline{\mathbb {R}^{d}_{+}})}. $$

On the other hand, from Corollary 6.2 we have

$$\|f\|^{2}_{L^{2}_{\beta}(\overline{\mathbb {R}^{d}_{+}})} \leq\frac{ (\int_{\Omega_{d+1}}\|(b,y)\|^{2s}|\widetilde{\mathcal{S}_{h}^{W}}f(b,y)|^{2} {\,d\mu_{\beta}(b,y)} )^{\frac{1}{2}} (\int_{\Omega _{d+1}}\|(b,y)\|^{2s}|{\mathcal{S}_{h}^{W}}f(b,y)|^{2} {\,d\mu_{\beta}(b,y)} )^{\frac{1}{2}}}{ C^{2}(s,s)\sqrt {\mathcal{C}_{h} ( {\mathcal{M}(e^{-y^{2}})(2t-4)} )}}. $$

The result immediately follows. □

## Conclusions

This paper is devoted to developing the localization operator theory for the continuous Weinstein wavelet transform. Given a symbol *a* and two Weinstein wavelets *h*, *k*, we investigate the multilinear mapping from $(a,h,k) \in L^{p}_{\mu_{\beta}}(\Omega _{d+1})\times L^{2}_{\beta}(\overline{\mathbb {R}^{d}_{+}})\times L^{2}_{\beta}(\overline{\mathbb {R}^{d}_{+}})$, $1 \leq p \leq\infty$, to the localization operator $\mathcal {L}_{h,k}(a)$, and we give sufficient conditions for $\mathcal {L}_{h,k}(a)$ to be bounded or to belong to a Schatten class. Our results are formulated in terms of time-frequency analysis.

The uncertainty principles in the context of the continuous Weinstein wavelet transform are also established.
